# Efficacy and acceptability of psychosocial interventions in asylum seekers and refugees: systematic review and meta-analysis

**DOI:** 10.1017/S2045796019000027

**Published:** 2019-02-11

**Authors:** G. Turrini, M. Purgato, C. Acarturk, M. Anttila, T. Au, F. Ballette, M. Bird, K. Carswell, R. Churchill, P. Cuijpers, J. Hall, L. J. Hansen, M. Kösters, T. Lantta, M. Nosè, G. Ostuzzi, M. Sijbrandij, F. Tedeschi, M. Valimaki, J. Wancata, R. White, M. van Ommeren, C. Barbui

**Affiliations:** 1 Cochrane Global Mental Health and WHO Collaborating Centre for Research and Training in Mental Health and Service Evaluation, Department of Neuroscience, Biomedicine and Movement Sciences, Section of Psychiatry, University of Verona, Verona, Italy; 2 Department of Psychology, Istanbul Sehir University, Orhantepe Mahallesi, Turgut Özal Bulvarı, Kartal/İstanbul, Turkey; 3 University of Turku, Faculty of Medicine, Turku, Finland; 4 Department of Mental Health and Substance Abuse, World Health Organization, Geneva, Switzerland; 5 Department of Dependence (SerD2), Azienda ULSS N. 8 Berica, Vicenza, Italy; 6 International Federation of Red Cross and Red Crescent Societies Reference Centre for Psychosocial Support/Danish Red Cross, Copenhagen, Denmark; 7 Centre for Reviews and Dissemination and Cochrane Common Mental Disorders Review Group, University of York, York, UK; 8 Department of Clinical and Developmental Psychology, Vrije Universiteit Amsterdam, Amsterdam Public Health Research Institute, Amsterdam, The Netherlands; 9 Department of Psychiatry II, Ulm University, Ulm, Germany; 10 Hong Kong Polytechnic University, Hong Kong, China SAR; 11 Department of Psychiatry and Psychotherapy, Division for Social Psychiatry, Medical University of Vienna, Vienna, Austria; 12 Institute of Psychology, Health and Society, University of Liverpool, Liverpool, UK

**Keywords:** Asylum seekers, mental health, psychosocial interventions, refugees

## Abstract

**Aims:**

In the past few years, there has been an unprecedented increase in the number of forcibly displaced migrants worldwide, of which a substantial proportion is refugees and asylum seekers. Refugees and asylum seekers may experience high levels of psychological distress, and show high rates of mental health conditions. It is therefore timely and particularly relevant to assess whether current evidence supports the provision of psychosocial interventions for this population. We conducted a systematic review and meta-analysis of randomised controlled trials (RCTs) assessing the efficacy and acceptability of psychosocial interventions compared with control conditions (treatment as usual/no treatment, waiting list, psychological placebo) aimed at reducing mental health problems in distressed refugees and asylum seekers.

**Methods:**

We used Cochrane procedures for conducting a systematic review and meta-analysis of RCTs. We searched for published and unpublished RCTs assessing the efficacy and acceptability of psychosocial interventions in adults and children asylum seekers and refugees with psychological distress. Post-traumatic stress disorder (PTSD), depressive and anxiety symptoms at post-intervention were the primary outcomes. Secondary outcomes include: PTSD, depressive and anxiety symptoms at follow-up, functioning, quality of life and dropouts due to any reason.

**Results:**

We included 26 studies with 1959 participants. Meta-analysis of RCTs revealed that psychosocial interventions have a clinically significant beneficial effect on PTSD (standardised mean difference [SMD] = −0.71; 95% confidence interval [CI] −1.01 to −0.41; *I*^2^ = 83%; 95% CI 78–88; 20 studies, 1370 participants; moderate quality evidence), depression (SMD = −1.02; 95% CI −1.52 to −0.51; *I*^2^ = 89%; 95% CI 82–93; 12 studies, 844 participants; moderate quality evidence) and anxiety outcomes (SMD = −1.05; 95% CI −1.55 to −0.56; *I*^2^ = 87%; 95% CI 79–92; 11 studies, 815 participants; moderate quality evidence). This beneficial effect was maintained at 1 month or longer follow-up, which is extremely important for populations exposed to ongoing post-migration stressors. For the other secondary outcomes, we identified a non-significant trend in favour of psychosocial interventions. Most evidence supported interventions based on cognitive behavioural therapies with a trauma-focused component. Limitations of this review include the limited number of studies collected, with a relatively low total number of participants, and the limited available data for positive outcomes like functioning and quality of life.

**Conclusions:**

Considering the epidemiological relevance of psychological distress and mental health conditions in refugees and asylum seekers, and in view of the existing data on the effectiveness of psychosocial interventions, these interventions should be routinely made available as part of the health care of distressed refugees and asylum seekers. Evidence-based guidelines and implementation packages should be developed accordingly.

## Introduction

Over the past two decades, the population of forcibly displaced migrants has grown substantially, from 33.9 million in 1997 to 65.6 million in 2016, of which 22.5 million people were refugees and 2.8 million asylum seekers (UNHCR, 2017). Most of this increase was driven by ongoing conflicts in Syria, Iraq, Yemen, as well as in sub-Saharan Africa, including Burundi, the Central African Republic, the Democratic Republic of the Congo, South Sudan and Sudan. Turkey and Pakistan have hosted the largest number of refugees worldwide, and Uganda experienced a dramatic increase in this population. The conflict in Syria dominated figures for newly recognised refugees in 2016 with 824 400 new recognitions, making this the most common country of origin, followed by Afghanistan (UNHCR, 2017).

Refugees are a subset of a wider population who are forcibly displaced, as the term refugee is a legal definition related to the 1951 United Nations Convention on the rights of refugees (United Nations General Assembly, 1951). Thus not all forcibly displaced migrants are recognised as refugees, and many may be asylum seekers or internally displaced people (IOM, 2011).

From a public mental health perspective, there is epidemiological evidence showing that exposure to extreme stressors, including major losses and potentially traumatic events such as torture and war exposure, are disproportionately experienced by refugees and asylum seekers before and during displacement (Bogic *et al*., 2012; Priebe *et al*., 2016). In addition, post-displacement stressors that are important for mental health include resettlement, language barriers and perceived stigma and discrimination (Kirmayer *et al*., 2011; Miller and Rasmussen, 2010). Consequently, as compared with the general population, refugees have been shown to experience considerably higher levels of psychological distress, higher levels of social distress in different domains (i.e. demographic, economic, neighbourhood, environmental events and social and cultural domains) (Lund *et al*., 2014; Lund *et al*., 2018), and higher rates of some mental health conditions, including post-traumatic stress disorder (PTSD), depression and anxiety, although findings are not consistent across studies (Turrini *et al*., 2017). As compared with the general population, psychosis has also been shown to be more frequent in people exposed to trauma and displacement (Close *et al*., 2016; Dapunt *et al*., 2017).

Randomised controlled trials (RCTs) suggest that psychosocial interventions, that is interventions with a focus on the interrelation between social circumstances and peoples’ thoughts, emotions and behaviours, may be helpful in treating some mental disorders in asylum seekers and refugees (Nosè *et al*., 2017). However, studies are heterogeneous, and existing reviews are narrative or focused on selected populations of asylum seekers or refugees, for example those with a formal diagnosis of PTSD, or only those displaced and resettled in particular settings only (Nosè *et al*., 2017; Tribe *et al*., 2017; Thompson *et al*., 2018). No reviews including asylum seekers and refugees with psychological distress have ever been conducted. While some guidance for the provision of psychosocial interventions exists (e.g. United Kingdom NICE guidelines for PTSD), specific evidence-based guidelines have yet to be developed for this population. It is therefore important and timely to assess whether current evidence supports the provision of psychosocial interventions for asylum seekers and refugees with psychological distress (Koesters *et al*., 2018). Therefore, the aim of this review was to ascertain the efficacy of psychosocial interventions on PTSD, depressive and anxiety outcomes in adults and children asylum seekers and refugees with psychological distress.

## Methods

The protocol for this review was registered in the International Prospective Register of Systematic Reviews (PROSPERO), registration number: CRD42017071523.

### Identification and selection of studies

The following bibliographical databases were searched up to September 2017: Cochrane Central Register of randomised trials (CENTRAL), CINAHL, EMBASE, PILOTS, PsycINFO, PubMed and Web of Science. The McMaster University algorithm to locate RCTs was used and complemented with the terms asylum seeker*, refugee*, migrant*, immigrant*, torture* AND psychother*, psychosocial, therapy, intervent*, treatment, counsel, support*, mental (both MESH terms and text words). Studies in any language were considered for inclusion. Grey literature was searched using the databases listed in the Cochrane Handbook for Systematic Reviews of Interventions (Higgins and Green, 2011). We reviewed the reference lists of key books and book chapters, and the reference lists of previously published reviews and original research articles were scrutinised to identify publications not covered by the original database searches. We also cross-checked the search performed by Cochrane on psychological therapies for the treatment of mental disorders in low- and middle-income countries (LMICs) affected by humanitarian crises (Purgato *et al*., 2015). Details of the search strategy and screening process are reported in online Supplementary Appendix 1. The selection process was recorded in agreement with the Preferred Reporting Items for Systematic Reviews and Meta-Analyses (PRISMA) and it was performed by two independent authors (Moher *et al*., 2009).

Studies meeting the following criteria were included: (a) RCTs; (b) assessing the efficacy of a psychosocial intervention aimed at reducing mental health problems; (c) reporting PTSD, depressive or anxiety outcomes measured with validated rating scales; (d) comparing psychosocial interventions with: treatment as usual (TAU), or no treatment, or waiting list (WL), or psychological placebo (non-manualised forms of person-centred support, e.g. supportive counselling); (e) including participants having an asylum seeker and/or refugee status and (f) being of any age and resettled in high-income countries (HIC) or in LMICs, as classified by the World Bank criteria (World Bank, 2018).

### Outcome measures

The primary outcomes were the mean scores at post-intervention on validated rating scales measuring PTSD, depressive and anxiety symptoms. For PTSD symptoms, data were extracted from the Clinician-Administered PTSD Scale (CAPS) (Blake *et al*., 1995) or Harvard Trauma Questionnaire (HTQ) (Mollica *et al*., 1992) or from any other PTSD rating scale with evidence of validity and reliability. For depressive symptoms, data were extracted from the Hamilton Depression Rating Scale (HDRS) (Hamilton, 1960) or Beck Depression Inventory-II (BDI-II) (Beck *et al*., 1996) or from any other depression rating scale with evidence of validity and reliability. For anxiety symptoms, data were extracted from the Hamilton Rating Scale for Anxiety (HRSA) (Hamilton, 1959) or from any other anxiety rating scale with evidence of validity and reliability. Secondary outcomes were the following: PTSD, depressive and anxiety symptoms at study follow-up; treatment acceptability, measured as the number of participants who dropped out during the trial by any cause; global functioning, and quality of life (the latter added post-hoc, as measured by any validated rating scale at post-treatment and follow-up).

For the purposes of this review, assessments occurring within 1 month after the delivery of the intervention were considered post-treatment measures, while assessments occurring more than 1 month after the delivery of the intervention were considered follow-up measures.

### Data extraction and quality assessment

Two review authors (GT and MP) independently extracted the data on participant characteristics, intervention details and outcome measures. Disagreements were resolved by discussion and consensus with a third member of the team (CB). Data extraction was performed in agreement with the Cochrane Handbook for Systematic Reviews of Interventions, Chapter 7 (Higgins and Green, 2011). For continuous outcomes, the mean scores at post-intervention or, if not available, the mean change from baseline, the standard deviation of these values, and the number of patients included in these analyses, were extracted. For dichotomous outcomes, the number of participants undergoing the randomisation procedure, and the number of patients leaving the study early for any reason, were recorded as a measure of treatment acceptability. For crossover studies, only data of the first period (before crossing over) were extracted. When outcome data were not reported, trial authors were contacted with a request to supply the missing information.

The risk of bias of included trials was assessed independently by two review authors (GT and FB) using the ‘Risk of bias’ assessment tool developed by Cochrane (Higgins and Green, 2011). This tool assesses possible sources of bias in clinical trials, including random sequence generation and allocation concealment (selection bias), blinding of participants and personnel (detection bias), blinding of outcome assessment (detection bias), incomplete outcome data (attrition bias), selective reporting of outcomes (reporting bias) and other biases (e.g. sponsorship bias). To determine the risk of bias of a trial, for each criterion we evaluated the presence of sufficient information and the likelihood of potential bias. We rated each criterion as ‘low risk of bias’, ‘high risk of bias’ or ‘unclear risk of bias’ (indicating either lack of information or uncertainty over the potential for bias) (Higgins and Green, 2011). If the raters disagreed, the final rating was made by consensus with the involvement (if necessary) of a third review author (CB). Details on the quality assessment process are given in online Supplementary Appendix 1.

### Data synthesis

Data were initially entered and analysed with Review Manager (RevMan; version, 5.3.5; Review Manager (RevMan) [Computer program]. Version 5.3., 2014), as recommended by the Cochrane Handbook (Higgins and Green, 2011), and then independently re-entered into a spreadsheet and analysed within the *metan* module in Stata 15.1 (StataCorp., 2017. Stata Statistical Software: Release 15, 2017). Statistical outputs were cross-checked for consistency. In accordance with recent efforts towards a data sharing culture (Barbui, 2016; Barbui *et al*., 2016), the spreadsheet with the full dataset is made available as part of this publication (online Supplementary Appendix 2).

For continuous outcomes, we pooled the standardised mean differences (SMDs) as different measurement scales were used. A loose intention-to-treat analysis was applied, whereby all participants with at least one post-baseline measurement were represented by their last observations carried forward (Higgins and Green, 2011). When only *p* or standard error values were reported, standard deviations were calculated according to Altman and Bland (1996, 2011). If standard deviations could not be calculated, they were imputed using validated methodology (Furukawa *et al*., 2006). Because some studies had relatively small sample sizes we corrected the effect size for small sample bias, using Hedges’ *g* (Higgins and Green, 2011). For dichotomous outcomes a Mantel–Haenszel risk ratio (RR) was calculated. Continuous and dichotomous outcomes were analysed using a random-effects model, with 95% confidence intervals (CI) (Higgins and Green, 2011). Studies that compared two or more formats of similar psychosocial interventions were included in meta-analysis by combining group arms into a single group, as recommended in section 16.5 of the Cochrane Handbook (Higgins and Green, 2011). When studies compared two or more different intervention groups were included separately, and the shared inactive intervention group was divided out approximately evenly among the comparisons. For dichotomous outcomes, both the number of events and the total number of patients were divided up. For continuous outcomes, only the total number of participants was divided up and the means and standard deviations left unchanged.

We calculated the *I*^2^-statistic, which quantifies the effect of statistical heterogeneity, providing a measure of the degree of inconsistency in the studies’ results in percentages (Higgins and Green, 2011). We calculated 95% CIs around *I*^2^ using the *heterogi* module in Stata 15.1 (Orsini *et al*., 2006).

To explore heterogeneity, the following subgroup analyses were performed: type of psychosocial intervention (narrative exposure therapy (NET) *v*. other cognitive behavioural therapies (CBT) *v*. eye movement desensitisation and reprocessing (EMDR) *v*. other types of intervention), age (adult *v*. children/adolescents/mixed), mental health condition at baseline (studies enrolling participants with a formal diagnosis of PTSD or depression or anxiety *v*. studies enrolling participants without a formal diagnosis at baseline), study setting (HICs *v*. LMICs), control condition (TAU/no treatment *v*. WL *v*. psychological placebo), level of intervention (individual intervention *v*. group intervention) and degree of risk of bias (high risk: more than one high or unclear risk items *v*. low risk: all other studies).

To investigate the impact of each study on the pooled effect, we consecutively removed each study as a possible outlier to test its impact on the combined effect, as implemented in comprehensive meta-analysis (CMA) (Borenstein *et al*., 2009).

Publication bias was tested with CMA by visually inspecting the PTSD funnel plot. Egger's test of the intercept was conducted to quantify the bias captured by the asymmetry of the funnel plot and to test whether it was significant.

To provide a measure of clinical significance, the number-needed-to-treat (NNT) was calculated for primary outcomes, which indicates the number of patients that would need to be treated in order to generate one additional positive outcome (Kraemer and Kupfer, 2006). We first transformed the SMDs for the primary outcomes into odds ratios (as implemented in CMA) and then we calculated NNTs assuming different control condition event rates. Additionally, in order to produce a tabular synoptic overview of the main review findings and quality, easily understandable for patients, policy makers, research planners, guideline developers and other stakeholders, data were summarised according to the methodology described by the GRADE working group (Guyatt *et al*., 2011). We followed the World Health Organization criteria for summarising and aggregating evidence (Barbui *et al*., 2010, 2015).

## Results

### Characteristics of included studies

**Fig. 1.**
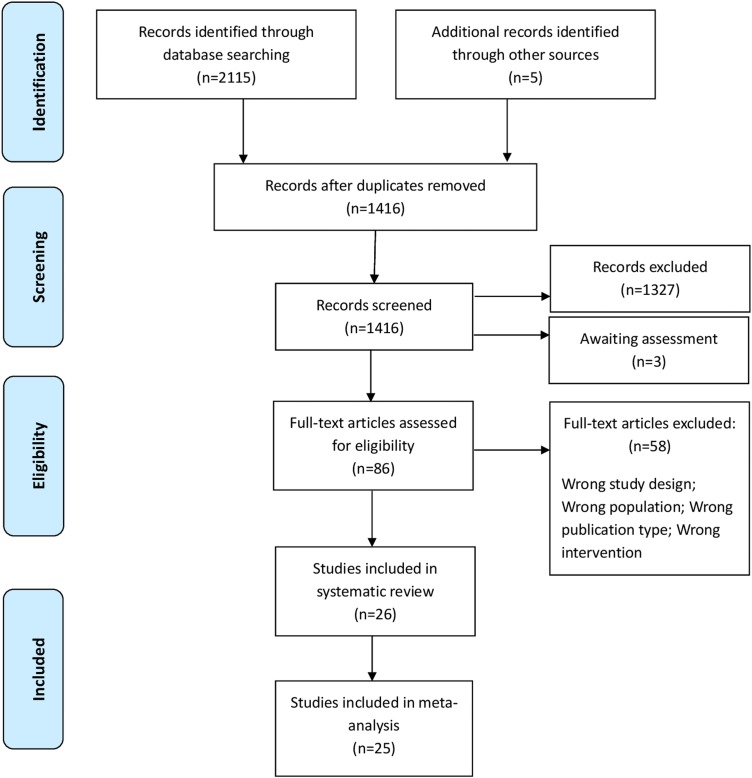
PRISMA flow-chart diagram.

The electronic search yielded a total number of 1416 records (after removal of duplicates). After title and abstract screening, 88 full text papers were considered for inclusion, of which 26 studies fulfilled the eligibility criteria and were included in the systematic review (Otto *et al*., 2003; Hinton *et al*., 2004, 2005, 2009; Neuner *et al*., 2004, 2008, 2010; Baker and Jones, 2006; Weine *et al*., 2008; Ruf *et al*., 2010; Adenauer *et al*., 2011; Liedl *et al*., 2011; Renner *et al*., 2011; Ter Heide *et al*., 2011, 2016; Kalantari *et al*., 2012; Stenmark *et al*., 2013; Bolton *et al*., 2014; Hijazi *et al*., 2014; Meffert *et al*., 2014; Morath *et al*., 2014; Acarturk *et al*., 2015, 2016; Buhmann *et al*., 2016; Ooi *et al*., 2016; Weinstein *et al*., 2016) (Fig. 1). References of excluded studies and reasons for exclusion are reported in online Supplementary Appendix 1.

Thirteen studies had a WL control condition, while 11 compared a psychosocial intervention with TAU or no treatment. In four studies psychosocial interventions were compared with a psychological placebo (non-manualised forms of person-centred support administered with the same/similar frequency, quantity and format as the experimental intervention). Psychological placebo interventions were: supportive counselling, trauma counselling and stabilisation therapy. The mean study sample size was 75 participants (range 10–347) (Table 1). Eighteen studies were conducted in HICs, and eight in LMICs. Twelve studies recruited participant samples that were homogeneous for nationality, while in the remaining studies a range of nationalities were represented in the study sample. Study participants were treatment-seeking in five studies, while in the remaining studies they were not actively treatment-seeking or they were referred by local organisations, social workers or general practitioners. Eighteen studies recruited participants with a formal diagnosis of a mental health condition (PTSD: 17 studies; comorbid PTSD and depression: one study) and the remaining studies recruited participants with psychological distress in the absence of formal assessment for mental disorder (Table 1).

**Table 1.** Selected characteristics of included studies

Study	Country	Ethnic group	Treatment (no. of sessions – intervention level)	Comparison group	*N*	Outcome measures	Mental health status at recruitment	Follow-up (months)	Allowed medication
Acarturk *et al*. (2015)	Turkey	Syria	EMDR (7 – I)	Waiting list (WL)	29	IES-R, BDI-II	PTSD symptoms	1	No
Acarturk *et al*. (2016)	Turkey	Syria	EMDR (4.2 – I)	WL	98	HTQ, BDI-II, HSCL-25	PTSD	1	No
Adenauer *et al*. (2011)	Germany	Middle and central east, the Balkans, Africa	NET (12 – I)	WL	44	CAPS, HDRS	PTSD, depression	4	Yes
Baker and Jones (2006)	Australia	Sudanese, Iranian, Liberian, Rwandan, Ethiopian, and Congolese	Music therapy (10 – G)	No treatment	31	BSI	Behavioural problems	Post-treatment	Unclear
Bolton *et al*. (2014)	Thailand	Burman, others (Karen, Kayah, Kachin, Mon, Chin, Rakhine, Shan)	CETA (10 – I)	WL	347	HTQ, HSCL-25	PTSD symptoms, depression	Post-treatment	Unclear
Buhmann *et al*. (2016)	Denmark	Iraq, Iran, Lebanon, Ex-Yugoslavia, Afghanistan, other	CBT (16 – I)	WL	138	HTQ, HRSD, HRSA, SDS	PTSD	Post-treatment	Yes
Hijazi *et al*. (2014)	USA	Iraq	NET (3 – I)	WL	63	HTQ, BDI-II, WHO-5	PTSD symptoms	3	Unclear
Hinton *et al*. (2004)	USA	Cambodia, Vietnam	CBT (11 – I)	WL	12	HTQ, HSCL-25	PTSD	Post-treatment	Yes
Hinton *et al*. (2005)	USA	Cambodia	CBT (12 – I)	TAU	40	CAPS, ASI	PTSD	Post-treatment	Yes
Hinton *et al*. (2009)	USA	Cambodia	CBT (12 – I)	TAU	24	CAPS	PTSD	Post-treatment	Yes
Kalantari *et al*. (2012)	Iran	Afghanistan	Writing for recovering (6 – G)	No treatment	64	TGIC	PTSD symptoms	Post-treatment	Unclear
Liedl *et al*. (2011)	Germany, Switzerland	Balkans, Turkey, Others	CBT-BF (10 – I)	WL *v*. CBT-BF + physical activity	36	PDS, HSCL-25	PTSD symptoms	3	Unclear
Meffert *et al*. (2014)	Egypt	Sudan	IPT (6 – I)	WL	22	HTQ, BDI-II, CTS	PTSD symptoms	Post-treatment	Unclear
Morath *et al*. (2014)	Germany	Africa, Middle East	NET (12 – I)	WL	34	CAPS, HAM-D	PTSD	4	Yes
Neuner *et al*. (2004) (1) (2)^a^	Uganda	Sudan	NET (4 – I)	TAU (1) *v*. psychological placebo (2) (supportive counselling)	43	PDS, SF-12	PTSD	12	Unclear
Neuner *et al*. (2008) (1) (2)^b^	Uganda	Somalia, Rwanda	NET (6 – I)	No treatment (1) *v*. psychological placebo (2) (trauma counselling)	277	PDS	PTSD	6	Unclear
Neuner *et al*. (2010)	Germany	Turkey, Balkans, Africa	NET (9 – I)	TAU	32	PDS, HSCL-25	PTSD	6	Yes
Ooi *et al*. (2016)	Australia	Africa, Asia, Middle East	TRT(CBT) (8 – G)	WL	82	CRIES-13, DSRS, SDQP	PTSD	Post-treatment	Unclear
Otto *et al*. (2003)	USA	Cambodia	CBT (10 – G)	No treatment	10	CAPS, SCL-90-R	PTSD	Post-treatment	Yes
Renner *et al*. (2011)	Austria	Chechnya	CROP (15 – G)	WL *v*. CBT *v*. EMDR	94	HTQ	PTSD symptoms	Post-treatment	Unclear
Ruf *et al*. (2010)	Germany	Turkey, Balkan, Syria, Chechnya, Russia, Georgia	KIDNET (8 – I)	WL	26	UCLA PTSD index	PTSD	12	Unclear
Stenmark *et al*. (2013)	Norway	Iraq , Afghanistan, other Middle East Countries , African countries, other	NET (10 – I)	TAU	81	CAPS, HAM-D	PTSD	6	Yes
Ter Heide *et al*. (2011)	Netherlands	Afghanistan, Algeria, Angola, Bosnia, Iran, Iraq, Lebanon and Turkey	EMDR (11 – I)	Psychological placebo (stabilisation therapy)	20	HTQ, HSCL-25, WHOQOL-BREF	PTSD	3	Yes
Ter Heide *et al*. (2016)	Netherlands	Iraq, Afghanistan, Ex-Yugoslavia, altri Paesi del Medio Oriente, Africa	EMDR (12 – I)	Psychological placebo (stabilisation therapy)	74	CAPS, HSCL-25, WHOQOL-BREF	PTSD	3	Yes
Weine *et al*. (2008)	USA	Bosnia	FGI (9 – G)	No treatment	197	PSS, CES-D	PTSD	18	Unclear
Weinstein *et al*. (2016)	Jordan	Syria	Need-satisfaction intervention (NA – I)	No treatment	41	PSS; CES-D	PTSD symptoms, depressive symptoms	Post-treatment	Unclear

I, individual; G, group; NET, narrative exposure therapy; EMDR, eye movement desensitisation and reprocessing; CETA, common elements treatment approach; CBT, cognitive behaviour therapy; CBT-BF, biofeedback-based cognitive behavioural intervention; IPT, interpersonal psychotherapy; TRT, teaching recovery techniques; CROP: culture-sensitive oriented peer; KIDNET, narrative exposure therapy for children; FGI, family-group intervention; TAU, treatment as usual; IES-R, impact of event scale-revised; BDI-II, Beck depression inventory-II; HTQ, Harvard trauma questionnaire; HSCL-25, Hopkins Symptoms Checklist-25; CAPS, Clinician-Administered PTSD Scale; HDRS, Hamilton Depression Rating Scale; BSI, behavioural symptom index; HARSD, Hamilton Rating Scale for Depression; HRSA, Hamilton Rating Scale for Anxiety; SDS, Sheehan Disability Scale; WHO-5, World Health Organization's Well-being Index; ASI, Anxiety Sensitivity Index; TGIC, Traumatic Grief Inventory for Children; PDS, Post Traumatic Stress Diagnostic scale; CTS, Conflict Tactics Scale; HAM-D, Hamilton Depression Rating Scale; SF-12, 12-item version of the Medical Outcome Study Self Report Form; CRIES-13, Children's Revised Impact of Event Scale; DSRS, Birleson Depression Self-Rating Scale; SDQP, parent-rated strengths and difficulties questionnaire; SCL-90, Symptom Checklist-90-R; UCLA PTSD index, UCLA; WHOQOL-BREF, The World Health Organization Quality of Life-BREF version; PSS, The PTSD Symptoms Scale; CES-D, The Center for Epidemiological Studies Depression Scale; NA, not applicable; PTSD, post-traumatic stress disorder.

a Three-arm study: (1) corresponds to comparison between NET *v*. TAU; (2) corresponds to comparison between NET *v*. psychological placebo.

b Three-arm study: (1) corresponds to comparison between NET *v*. no treatment; (2) corresponds to comparison between NET *v*. psychological placebo.

The following interventions were included: NET, a manualised short-term variant of CBT with a trauma focus (seven studies), Narrative Exposure Therapy for children (KIDNET), a form of NET adapted for children (one study), EMDR (four studies), music therapy (one study), Common Elements Treatment Approach (CETA) (one study), CBT (six studies), writing for recovery (one study), interpersonal psychotherapy (IPT) (one study), Teaching Recovery Techniques (TRT), a form of CBT (one study), Culture-Sensitive Oriented Peer (CROP) (one study), Family-Group Intervention (FGI) (one study), need-satisfaction intervention (one study). The quality of the studies varied as 16 of the 26 studies included in the primary outcome analysis were at high risk of bias in two or three items of the Cochrane risk of bias tool (online Supplementary Appendix 1).

### Efficacy of psychosocial interventions: primary outcomes

**Fig. 2.**
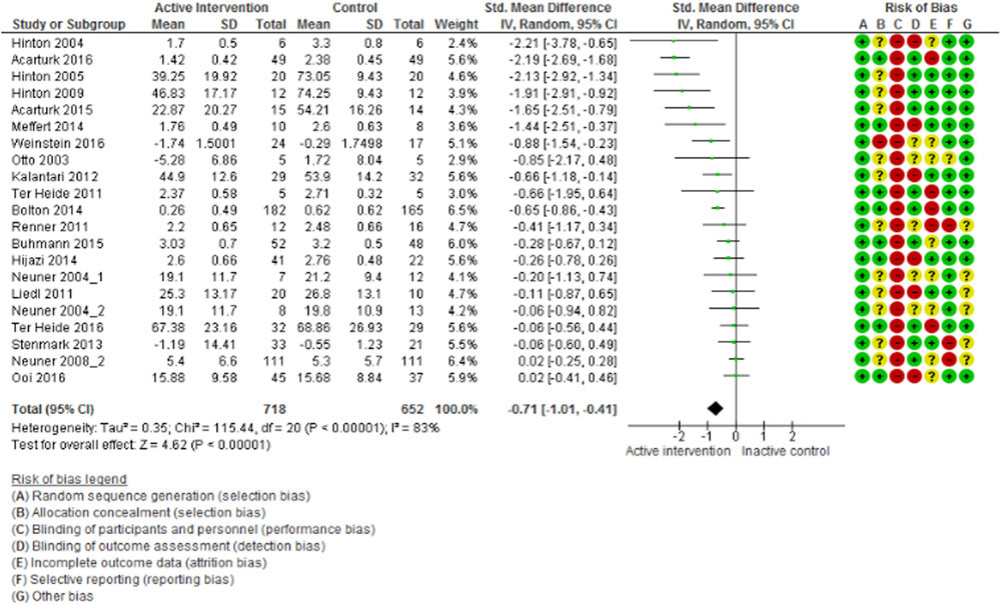
Efficacy of psychosocial interventions in refugees and asylum seekers: PTSD symptoms post intervention.

The meta-analysis of PTSD outcomes (20 studies, 1370 participants) showed that psychosocial interventions were effective in decreasing PTSD symptoms relative to controls (SMD = −0.71; 95% CI −1.01 to −0.41; *I*^2^ = 83%; 95% CI 78–88; GRADE certainty in estimate: moderate) (Fig. 2). Visual inspection of the funnel plot and Egger's test (*p* = 0.12) did not suggest publication bias (online Supplementary Appendix 1). Removing each of the studies as a possible outlier did not change the overall estimate. Removing the five studies with outlier results (Hinton *et al*., 2004, 2005, 2009; Acarturk *et al*., 2015, 2016) the overall estimate remained significant (SMD = −0.33; 95% CI −0.52 to −0.14; *I*^2^ = 49%; 95% CI 10–72) with heterogeneity below 50%.

**Fig. 3.**
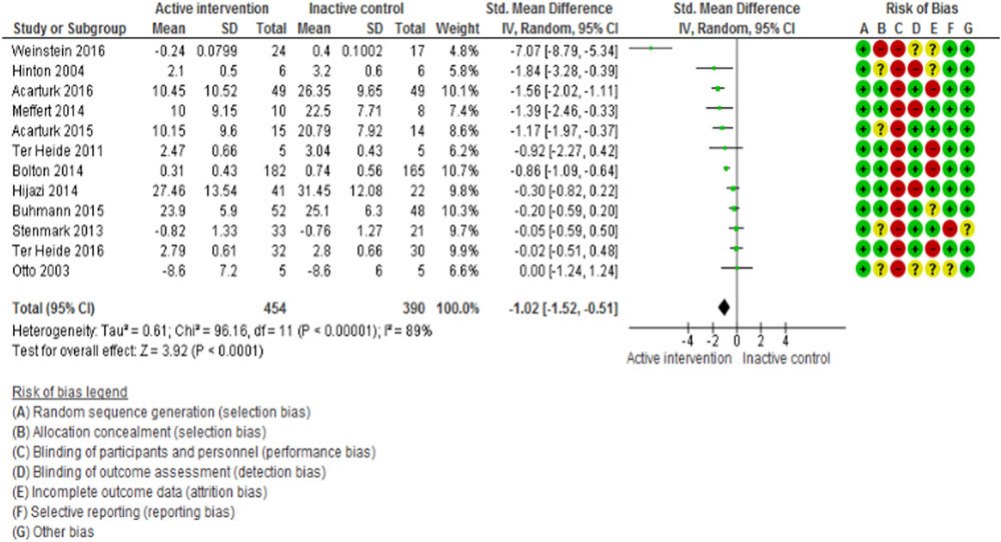
Efficacy of psychosocial interventions in refugees and asylum seekers: depressive symptoms post intervention.

**Fig. 4.**
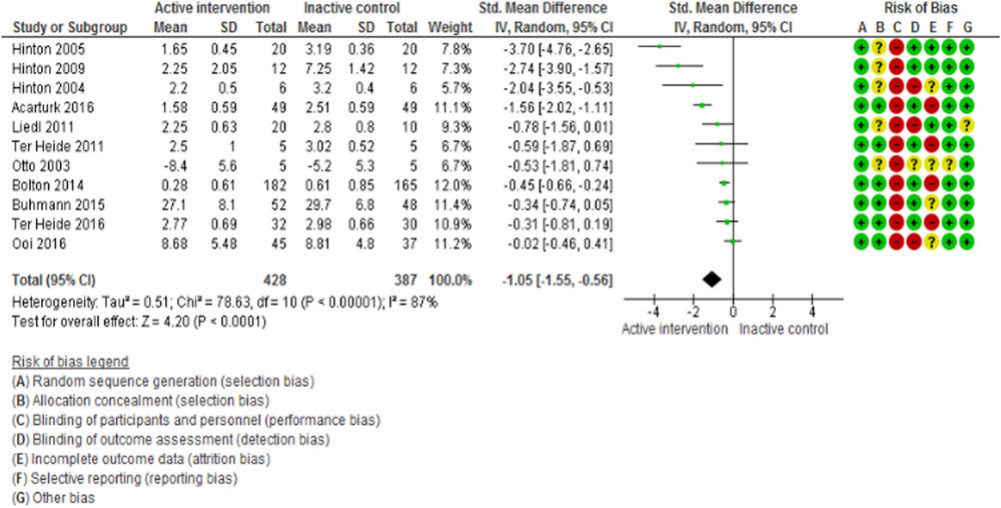
Efficacy of psychosocial interventions in refugees and asylum seekers: anxiety symptoms post intervention.

The meta-analysis of depression (12 studies, 844 participants) and anxiety (11 studies, 815 participants) outcomes showed that psychosocial interventions were effective in decreasing depressive symptoms (SMD = −1.02; 95% CI −1.52 to −0.51; *I*^2^ = 89%; 95% CI 82–93; GRADE certainty in estimate: moderate) and anxiety symptoms (SMD = −1.05; 95% CI −1.55 to −0.56; *I*^2^ = 87%; 95% CI 79–92; GRADE certainty in estimate: moderate) relative to controls (Fig. 3 and 4).

### Efficacy of psychosocial interventions: secondary outcomes

The efficacy of psychosocial interventions was maintained at follow-up assessments for PTSD outcomes (11 studies, 711 participants; SMD = −1.08; 95% CI −1.81 to −0.35; *I*^2^ =  83%; 95% CI 72–89), depressive outcomes (eight studies, 371 participants; SMD = −1.28; 95% CI −2.27 to −0.30; *I*^2^ = 88%; 95% CI 79–93) and anxiety outcomes (three studies, 171 participants; SMD = −0.49; 95% CI −0.93 to −0.05; *I*^2^ = 70%; 95% CI 0–91). In terms of treatment acceptability, we found that psychosocial interventions were not associated with more participants leaving the study early than the control condition (23 studies, 1636 participants; RR = 0.96; 95% CI 0.82–1.13; *I*^2^ = 0%; 95% CI 0–52).

In terms of functioning (four studies, 547 participants; SMD = −0.17; 95% CI −0.58 to 0.24; *I*^2^ = 74%; 95% CI 27–91) and quality of life (five studies, 173 participants; SMD =  0.23; 95% CI −0.08 to 0.54; *I*^2^ = 0%; 95% CI 0–79), psychosocial interventions were not different from control interventions (Table 2).

**Table 2.** Meta-analyses of secondary outcomes

Meta-analysis	Comparisons (*N*)	Patients (*N*)	SMD^a^	95% CI	*I*^2^ (%)	95% CI	*p*
PTSD (follow-up)	13	711	−1.08	−1.81 to −0.35	83	72–89	0.004
Depression (follow-up)	8	371	−1.28	−2.27 to −0.30	88	79–93	0.010
Anxiety (follow-up)	3	171	−0.49	−0.93 to −0.05	70	0–91	0.030
Drop-out rate (RR)	25	1636	0.96	0.82 to 1.13	0	0–52	0.620
Functioning (post-treatment)	4	547	−0.17	−0.58 to 0.24	74	27–91	0.420
Functioning (follow-up)	1	25	−0.81	−1.63 to 0.01	NA	NA	0.050
Quality of life (post-treatment)^b^	5	173	0.23	−0.08 to 0.54	0	0–79	0.140
Quality of life (follow-up)^b^	5	174	0.27	−0.08 to 0.63	17	0–83	0.130

*N*, number; SMD, standardised mean difference; CI, confidence interval; PTSD, post-traumatic stress disorder.

a Negative values favour active interventions.

b Positive values favour active interventions.

### Subgroup analysis

The type of psychosocial intervention was significantly associated with overall efficacy. While CBT was effective in decreasing PTSD and anxiety symptoms, EMDR was effective in terms of depressive symptoms only, and NET failed to show a significant effect (online Supplementary Appendix 1). In terms of delivery modality, no differences were observed between individual and group interventions (online Supplementary Appendix 1). The type of control condition was significantly associated with overall efficacy. While psychosocial interventions were effective against WL, TAU or no treatment, no difference was found against psychological placebo (online Supplementary Appendix 1). Subgroup analysis by age revealed that most studies were conducted in adults or in mixed populations of adults and children, and so there is uncertainty on the efficacy of psychosocial interventions in children in this population. The type of mental health condition was not associated with overall efficacy, thus suggesting the efficacy of these interventions both in participants with a formal diagnosis of a mental health condition and in those with psychological distress in absence of formal assessment for mental disorder (online Supplementary Appendix 1). In terms of country income level, studies conducted in HIC and studies conducted in LMIC provided similar findings for PTSD and anxiety outcome. For depressive outcomes, the efficacy of psychosocial interventions was more evident in studies conducted in LMICs (online Supplementary Appendix 1). Subgroup analysis by study quality revealed that psychosocial interventions were effective in terms of PTSD, depressive and anxiety outcomes in the subgroup of studies at high risk of bias, while in studies at low risk of bias psychosocial interventions were effective only in terms of PTSD outcomes (online Supplementary Appendix 1). Heterogeneity remained substantial in the majority of subgroups (online Supplementary Appendix 1).

### Number needed to treat

For the primary outcomes, meta-analysis results were transformed into NNTs. The provision of psychosocial interventions was associated with NNTs ranging between 2 and 3 in case of very high frequency of unfavourable outcomes in those receiving control conditions (60%), between 3 and 4 in case of high frequency of unfavourable outcomes in those receiving control conditions (40%), between 6 and 7 in case of moderate frequency of unfavourable outcomes in those receiving control conditions (20%), and between 12 and 14 in case of low frequency of unfavourable outcomes in those receiving control conditions (10%) (online Supplementary Appendix 1).

## Discussion

To our knowledge, this is the most comprehensive systematic review and meta-analysis conducted to date on the efficacy and acceptability of psychosocial interventions in asylum seekers and refugees. We found moderate quality evidence that psychosocial interventions have a beneficial effect on PTSD, depressive and anxiety symptoms, and results did not change in subgroup analyses. In addition, this beneficial effect was maintained at follow-up (measured at least one month after completion of the intervention), which is particularly relevant for populations exposed to ongoing post-migration stressors. Depending on the natural course of psychological distress in different target populations of asylum seekers and refugees, the magnitude of effect may reach a NNT between two and three in people showing low frequency of spontaneous improvement. This suggests that between two and three refugees and asylum seekers need to be treated in order for one to benefit; by contrast, in people showing high frequency of spontaneous improvement, the NNTs may be between 12 and 14. It is important to acknowledge that this variability depends on factors still largely unknown, particularly the expected improvement rate in those not receiving treatment. Another source of expected variability is related to the between-study heterogeneity observed, that likely depends on differences in populations, interventions, control conditions and contextual factors.

Despite the current emphasis on general well-being in studies involving this population, functioning and quality of life were as assessed in four studies only and no difference between psychosocial interventions and control conditions emerged.

In terms of type of psychosocial intervention, CBT with a trauma focus, EMDR and stress management interventions are generally recommended in the general population of adults with PTSD (Tol *et al*., 2013, 2014). This review, considering that the included interventions based on CBT might have a trauma-focused component, is in agreement with these recommendations except for EMDR, which failed to show a significant effect for PTSD as an outcome, although the CI around the point estimate did not exclude the possibility of a clinically relevant benefit. Given that only four studies on EMDR were included, we propose that current evidence base on EMDR in this specific population needs to be expanded. Another challenging finding was that NET, a manualised short-term variant of trauma-focused CBT, failed to show a beneficial effect for PTSD and depression outcomes at post-intervention. These findings are not consistent with the previous systematic review by Nosè *et al.*, which showed efficacy of NET for PTSD experienced by asylum seekers and refugees (Nosè *et al*., 2017). However, the Nosè *et al*. review focused on asylum seekers and refugees resettled in HIC only, and included non-randomised studies in addition to RCTs. Another aspect that may be relevant for NET is that some studies employed a psychological placebo, which is theoretically inactive. When using psychological placebo there may be factors such as a positive relationship with the therapist, which may be beneficial in terms of PTSD, depression and anxiety outcomes, possibly explaining the lack of efficacy of NET in this analysis. This is interesting in view of the existing debate on whether, and to what extent, the effects of psychosocial interventions are actually based on common factors (e.g. therapeutic alliance, therapist fidelity to therapeutic model) (Wampold, 2015). Methodologically, this finding would suggest that studies aiming to ascertain the true efficacy of psychosocial interventions should employ a psychological placebo rather than a no treatment or wait-list condition.

The efficacy of psychosocial interventions in children and adolescent refugees needs to be ascertained (Fazel, 2018). Unfortunately, only one study (Ruf *et al*., 2010) included in the current review was conducted with children from 7 years, with a few additional studies providing sparse data on adolescents. So far the best evidence for this population comes from a recent systematic review that included individual participant data of more than 3000 children exposed to traumatic events in humanitarian settings (Purgato *et al*., 2018). Assuming that the experience of displacement is not too dissimilar to the experience of migration (UNHCR, 2006), the finding that trauma focused CBT provided clinically relevant beneficial effects may consistently complement for children the findings on adults from the present review.

The present review has some limitations. A first limitation is that despite refugees and asylum seekers may be vulnerable to serious mental health conditions including psychosis, only PTSD, depression and anxiety outcomes were considered. We made this choice as these are the best studied mental health outcomes in this population, while data for psychosis are still too limited to be re-analysed to generate meaningful pooled estimates. Second, a limited number of studies were included, with a relatively low total number of participants contributing to the primary analysis, and an overall moderate to low quality of the included studies. In particular, lack of blinding emerged as a major issue as it may have increased risk of performance bias. However, detection bias should not have been substantially affected by lack blinding, as studies employed masked outcome assessors. A third concern is that follow-up assessments were not very long, leaving uncertainty on the long-term effect of psychological interventions. Another limitation is that the included studies differed with respect to background origins of the included populations, time since resettlement, year and country of study publication, outcome measures, content and modalities of delivering psychosocial interventions. All these differences likely contributed to the very high level of statistical heterogeneity that was detected when all studies were pooled together. Heterogeneity was not fully explained even by subgroup analyses, however the effects were generally consistent across them. Unfortunately, it was not possible to conduct subgroup analyses to investigate the role of some important variables, such as time since resettlement, the legal condition of the included population (refugees *v*. asylum seekers), and the use of pharmacological treatment.

All these limitations should be contextualised to the population and setting under study, as conducting research with refugees and asylum seekers is challenging. Potential participants may have limited command of the language of the host country, which complicates psychosocial treatments and obtaining informed consent for participating in research. Moreover, potential participants may not fully understand the health care system, the rules governing research and the reasons for being offered both a psychosocial intervention and participation in a research trial (Sijbrandij, 2018).

Any lack of adaptation and testing of the cultural appropriateness of interventions and measures might weaken the accuracy of the studies’ conclusions, taking into consideration that existing research showed that more extensive cultural adaptation of the interventions may be associated with larger effect sizes (Bass *et al*., 2007; Harper Shehadeh *et al*., 2016). Socio-cultural differences in relation to the psychological suffering exist, and the transposition of psychosocial interventions and models from Western to non-Western cultures, with very different understandings and ways of dealing with psychological distress, might potentially influence therapeutic relationship and outcomes (Barbui *et al*., 2017). Moreover, even though we were able to collect information about some basic therapists’ characteristics, details on therapists’ language and nationality, social/economic class, education, geography, age and background were very rarely reported. These characteristics might have an influence in the establishment of relationship and trust on the study outcomes (Kaiser *et al*., 2015; Haroz *et al*., 2017).

Given the pressing mental health needs of asylum seekers and refugees, and in view of the existing data on the effectiveness of psychosocial interventions, these forms of interventions should be made routinely available to distressed adults and children asylum seekers and refugees resettled in countries irrespective of (high-, middle- and low-) income category, also recognising that forms of supportive counselling (psychological placebo) may provide some initial relief in the short term, when access to mental health services may still be limited. In fact, the feasibility and sustainability of the availability of psychosocial interventions, especially in the long-term and especially in LMICs, may be an important challenge. To facilitate availability, brief, basic, group and non-specialist-delivered versions of these evidence-based psychosocial treatments should be considered as an affordable, scalable alternative. They are currently under investigation in several countries and early results from RCTs showed efficacy (Rahman *et al*., 2016; van't Hof *et al*., 2018).

In this review psychosocial interventions have been shown to provide clinically relevant beneficial effects not only in those with a diagnosis of a mental health condition but also in those with psychological distress without a formal assessment of mental disorder. However, since studies conducted in participants with psychological distress did not systematically exclude those with a diagnosis of mental disorder, the present review cannot be used to claim a preventative effect. To claim a preventative effect, future studies should be designed to include participants with psychological distress but without a mental disorder at study entry. Research should also aim to identify important mechanisms of change for these interventions e.g. improvement in functioning, increased hope, and/or enhanced quality of life.

In conclusion, this review provided evidence in support of the availability of psychological interventions with a trauma focus to refugees and asylum seekers. Specific evidence-based guidelines and implementation packages should be developed accordingly (Giacco and Priebe, 2018). Guidelines should be applicable to different social and health care organisations, and should be implemented to ensure that all people have equitable access to high-quality mental health care.

## Supplementary material

For supplementary material accompanying this paper visit https://doi.org/10.1017/S2045796019000027. click here to view supplementary material

## Data

Data supporting our findings are available in online Supplementary Appendix 1 and online Supplementary Appendix 2 (Dataset).
