# Supplementary material for: Efficacy and acceptability of psychosocial interventions in asylum seekers and refugees: systematic review and meta-analysis
Source: Epidemiol Psychiatr Sci. 2019 Feb 11;28(4):376–88. doi: 10.1017/S2045796019000027 (PMC6669989; doi:10.1017/S2045796019000027)
Supplement: Supplementary file 1 [file S2045796019000027sup.zip › S2045796019000027sup001/Appendix_1_revised.docx]

**Online supplemental material**

| 1. Search strategy............................................................................... | p. 2 |
| --- | --- |
| 1. List of included, excluded and awaiting studies............................ | p. 5 |
| 1. Risk of bias graph and summary..................................................... | p. 15 |
| 1. Risk of bias: review authors’ judgments about each risk of bias item for each included study………………………………………………..……. 2. Subgroup analyses.......................................................................... 3. Forest plots of secondary analyses................................................ | p. 16  p. 33  p. 37 |
| 1. Forest plots of subgroup analyses.................................................. | p. 40 |
| 1. Number needed to treat (NNT)………………………………………………….. | P. 53 |
| 1. GRADE tables................................................................................. | p. 54 |
| 1. Funnel plot..................................................................................... 2. Impact of each study on the pooled effect…………………………………. | p. 56  p. 57 |
| 1. PRISMA checklist............................................................................ | p. 58 |

**SEARCH STRATEGY**

| **PUBMED/MEDLINE** | |
| --- | --- |
| Search psychother* OR psychological OR psychosocial OR therapy OR intervent* OR treatment OR counsel* OR support* OR mental |  |
| Search (refugee* OR asylum seeker* OR migrant OR immigrant OR torture) |  |
| Search randomized controlled trial[Publication Type] OR (randomized[Title/Abstract] AND controlled[Title/Abstract] AND trial[Title/Abstract]) |  |
| Search (((psychother* OR psychological OR psychosocial OR therapy OR intervent* OR treatment OR counsel* OR support* OR mental)) AND ((refugee* OR asylum seeker*OR migrant OR immigrant OR torture))) AND (randomized controlled trial[Publication Type] OR (randomized[Title/Abstract] AND controlled[Title/Abstract] AND trial[Title/Abstract])) Filters: Clinical Trial | **285** |
|  | |
| **PsycINFO** | |
| (pub(randomized controlled trial) OR ab(randomized) AND ab(controlled) AND ab(trial)) AND (ab(refugee*) OR ab(asylum seeker*) OR ab(migrant) OR ab(immigrant) OR ab(torture)) AND (ab(psychother*) OR ab(psychological) OR ab(psychosocial) OR ab(therapy) OR ab(intervent*) OR ab(treatment) OR ab(counsel*) OR ab(support*) OR ab(mental)) | **82** |
| **CINHAL** | |
| S1: AB randomized controlled trial OR AB randomized AND AB controlled AND AB trial |  |
| S2: AB refugee* OR AB asylum seeker* OR AB migrant OR AB immigrant OR AB torture |  |
| S3: AB pharmacolog* OR AB psychother* OR AB psychological OR AB psychosocial OR AB therapy OR AB intervent* OR AB treatment OR AB counsel* OR AB support* OR AB mental |  |
| ((AB psychother* OR AB psychological OR AB psychosocial OR AB therapy OR AB intervent* OR AB treatment OR AB counsel* OR AB support* OR AB mental) AND (S1 AND S2 AND S3)) AND (S1 AND S2 AND S3) | **171** |
| **PILOTS** |  |
| (ab(trial) OR ab(randomize*) OR ab(randomise*)) AND (ab(refugee*) OR ab(asylum seeker*) OR ab(migrant) OR ab(immigrant) OR ab(torture)) AND (ab(psychother*) OR ab(psychological) OR ab(psychosocial) OR ab(therapy) OR ab(intervent*) OR ab(treatment)) | **64** |
| **CENTRAL** |  |
| #1"intervention":ti,ab,kw (Word variations have been searched), "treatment":ti,ab,kw (Word variations have been searched),"psychological":ti,ab,kw (Word variations have been searched),"psychosocial":ti,ab,kw (Word variations have been searched), "psychotherapy":ti,ab,kw (Word variations have been searched) |  |
| #2"refugee":ti,ab,kw (Word variations have been searched),"asylum":ti,ab,kw (Word variations have been searched), "migrant":ti,ab,kw (Word variations have been searched),"immigrant":ti,ab,kw (Word variations have been searched), "torture":ti,ab,kw (Word variations have been searched) |  |
| #3"randomised controlled clinical trials":ti,ab,kw (Word variations have been searched),"randomisation":ti,ab,kw (Word variations have been searched),trial":ti,ab,kw (Word variations have been searched) |  |
| #1 AND #2 AND #3 | **410** |
| **Web of Science** | |
| #1 TOPIC: (randomized controlled trial) OR TOPIC: (randomized) AND TOPIC: (controlled) AND TOPIC: (trial) |  |
| #2 TOPIC: (refugee*) *OR* TOPIC: (asylum seeker*) *OR* TOPIC: (migrant) *OR* TOPIC: (immigrant) *OR* TOPIC: (torture) |  |
| #3 TOPIC: (psychother*) OR TOPIC: (psychological) OR TOPIC: (psychosocial) OR TOPIC: (therapy) OR TOPIC:(intervent*) OR TOPIC: (treatment) OR TOPIC: (counsel*) OR TOPIC: (support*) OR TOPIC: (mental) |  |
| #1 AND #2 AND #3 | **788** |
| **EMBASE** | |
| #1 (psychother* OR psychological OR psychosocial OR therapy OR intervent* OR treatment OR counsel* OR support* OR mental) |  |
| #2 (refugee* OR asylum seeker*OR migrant OR immigrant OR torture) |  |
| #3 randomized controlled trial OR (randomized AND controlled AND trial) |  |
| #1 AND #2 AND #3 | **315** |
| TOTAL search results  TOTAL search results after removing duplicates | **2115**  **1416** |

**GREY LITERATURE**

We searched sources of grey literature, including dissertations and theses, reports, evaluations published on websites, and clinical guidelines and reports from regulatory agencies (when appropriate). In addition, we searched key agencies and initiatives in this field for relevant reports. Reference lists of all included studies and previous systematic reviews were checked for published reports and citations of unpublished research.

The following databases were searched: OpenSIGLE, The Healthcare Management Information Consortium (HMIC) database and PsycEXTRA.

**DIFFERENCES BETWEEN PROTOCOL AND REVIEW**

We post-hoc added quality of life as secondary outcome, measured by any validated rating scale at post-treatment and follow-up.

Moreover, we carried out the following additional subgroup analyses, not mentioned in the protocol:

- by control condition (treatment as usual (TAU)/no treatment vs. waiting list (WL) vs. psychological placebo)
- by level of intervention (individual intervention vs. group intervention)
- by risk of bias (high risk: more than one high or unclear risk items vs. low risk: all other studies).

**INCLUDED STUDIES**

| REFERENCES OF INCLUDED STUDIES |
| --- |
| 1. Acarturk C, Konuk E, Cetinkaya M, Senay I, Sijbrandij M, Cuijpers P, Aker T (2015). EMDR for Syrian refugees with posttraumatic stress disorder symptoms: results of a pilot randomized controlled trial. *European Journal of Psychotraumatology*; 6:27414. |
| 1. Acarturk C, Konuk E, Cetinkaya M, Senay I, Sijbrandij M, Gulen B, Cuijpers P (2016). The efficacy of eye movement desensitization and reprocessing for post-traumatic stress disorder and depression among Syrian refugees: results of a randomized controlled trial. *Psychological Medicine;* 46(12): 2583. |
| 1. Adenauer H, Catani C, Gola H, Keil J, Ruf M, Schauer M, Neuner F (2011). Narrative exposure therapy for PTSD increases top-down processing of aversive stimuli-evidence from a randomized controlled treatment trial. *BMC Neuroscience*;12(127):1–13. |
| 1. Baker F, Jones C (2006). The effect of music therapy services on classroom behaviours of newly arrived refugee students in Australia—a pilot study. *Emotional and Behavioural Difficulties*; 11(4), 249-260. |
| 1. Bolton P, Lee C, Haroz EE, Murray L, Dorsey S, Robinson C, Ugueto AM, Bass J(2014). A transdiagnostic community-based mental health treatment for comorbid disorders: development and outcomes of a randomized controlled trial among Burmese refugees in Thailand. *PLoS Medicine*; 11(11):e1001757. |
| 1. Buhmann CB, Nordentoft M, Ekstroem M, Carlsson J, Mortensen EL (2016). The effect of flexible cognitive-behavioural therapy and medical treatment, including antidepressants on post-traumatic stress disorder and depression in traumatised refugees: pragmatic randomised controlled clinical trial. *The British Journal of Psychiatry*; bjp.bp.114.150961. |
| 1. Hijazi AM, Lumley MA, Ziadni MS, Rapport LJ, Arnetz BB (2014). Brief narrative exposure therapy for posttraumatic stress in Iraqi refugees: a preliminary randomized clinical trial. *Journal of Traumatic Stress*; 27:314–22. |
| 1. Hinton DE, Hofmann SG, Pollack MH, Otto MW (2009). Mechanisms of efficacy of CBT for Cambodian refugees with PTSD: Improvement in emotion regulation and orthostatic blood pressure response. *CNS neuroscience & therapeutics*; 15(3), 255-263. |
| 1. Hinton DE, Chhean D, Pich V, Safren SA, Hofmann SG, Pollack MH (2005). A randomized controlled trial of cognitive-behavior therapy for Cambodian refugees with treatment-resistant PTSD and panic attacks: A cross-over design. *Journal of Traumatic Stress*; 18:617– 629. |
| 1. Hinton DE, Pham T, Tran M, Safren SA, Otto MW, Pollack MH (2004).CBT for Vietnamese refugees with treatment-resistant PTSD and panic attacks: a pilot study. *Journal of Traumatic Stress;* 17(5): 429-33. |
| 1. Kalantari M, Yule W, Dyregrov A, Neshatdoost H, Ahmadi SJ (2012). Efficacy of writing for recovery on traumatic grief symptoms of Afghani refugee bereaved adolescents: A randomized control trial. *OMEGA-Journal of death and dying;* 65(2): 139-150 |
| 1. Liedl A, Muller J, Morina N, Karl A, Denke C, Knaevelsrud C (2011) Physical activity within a CBT intervention improves coping with pain in traumatized refugees: results of a randomized controlled design. *Pain;* 12(2): 234–45. |
| 1. Meffert SM, Abdo AO, Alla OAA, Elmakki YOM, Omer AA, Yousif S, Metzle TJ, Marmar CR (2014). A pilot randomized controlled trial of interpersonal psychotherapy for Sudanese refugees in Cairo, Egypt. *Psychological Trauma: Theory, Research, Practice, and Policy*; 6(3): 240. |
| 1. Morath J, Gola H, Sommershof A, Hamuni G, Kolassa S, Catani C, Adenauer H, Ruf-Leuschner M, Schauer M, Elbert T, Groettrup M, Kolassa IT (2014). The effect of trauma-focused therapy on the altered T cell distribution in individuals with PTSD: evidence from a randomized controlled trial. *Journal of Psychiatric Research;* 54: 1–10. |
| 1. Neuner F, Kurreck S, Ruf M, Odenwald M, Elbert T, Schauer M (2010). Can asylum seekers with posttraumatic stress disorder be successfully treated? A randomized controlled pilot study. *Cognitive Behaviour Therapy;* 39(3):81–91   Secondary publication:  Schauer M, Elbert T, Gotthardt S, Rockstroh B, Odenwald M, Neuner F (2006). Imaginary reliving in psychotherapy modifies mind and brain. Verhaltenstherapie; 16 (2):96–103. |
| 1. Neuner F, Schauer M, Klaschik C, Karunakara U, Elbert T (2004). A comparison of narrative exposure therapy, supportive counseling, and psychoeducation for treating posttraumatic stress disorder in an african refugee settlement. *Journal of consulting and clinical psychology;* 72(4): 579. |
| 1. Neuner F, Onyut PL, Ertl V, Odenwald M, Schauer E, Elbert T (2008). Treatment of posttraumatic stress disorder by trained lay counselors in an African refugee settlement: a randomized controlled trial. *Journal of consulting and clinical psychology;* 76(4): 686. |
| 1. Ooi CS, Rooney RM, Roberts C, Kane RT, Wright B, Chatzisarantis N (2016). The efficacy of a group cognitive behavioral therapy for war affected young migrants living in Australia: A cluster randomized controlled trial. *Front Psychol* ; 7:1641. |
| 1. Otto MW, Hinton D, Korbly NB, Chea A, Phalnarith B, Gershuny BS, Pollack MH (2003). Treatment of pharmacotherapy-refractory posttraumatic stress disorder among Cambodian refugees: A pilot study of combination treatment with cognitive-behavior therapy vs. sertraline alone. *Behaviour Research and Therapy;* 41:1271–1276. |
| 1. Renner W, Bänninger-Huber E, Peltzer K (2011). Culture-Sensitive and Resource Oriented Peer (CROP)- Groups as a community based intervention for trauma survivors: A randomized controlled pilot study with refugees and asylum seekers from Chechnya. *Australasian Journal of Disaster and Trauma Studies;* 1: 1–13 |
| 1. Ruf M, Schauer M, Neuner F, Catani C, Schauer E, Elbert T (2010). Narrative exposure therapy for 7‐to 16‐year‐olds: A randomized controlled trial with traumatized refugee children. *Journal of traumatic stress;* 23(4): 437-445. |
| 1. Stenmark H, Catani C, Neuner F, Elbert T, Holen A (2013). Treating PTSD in refugees and asylum seekers within the general health care system. A randomized controlled multicenter study. *Behaviour Research and Therapy;* 51:641–7.   Secondary publication:  Halvorsen JØ, Stenmark H, Neuner F, Nordahl HM (2014). Does dissociation moderate treatment outcomes of narrative exposure treatment for PTSD? A secondary analysis from a randomized controlled clinical trial. Behaviour Research and Therapy; 57:21–8. |
| 1. Ter Heide FJ, Mooren TM, van de Schoot R, de Jongh A, Kleber RJ (2016).Eye movement desensitisation and reprocessing therapy v. stabilisation as usual for refugees: randomised controlled trial.*The British Journal of Psychiatry;*  bjp.bp.115.167775. |
| 1. ter Heide JJ, Mooren TM, Kleijn W, de Jongh A, Kleber RJ (2011). EMDR versus stabilisation in traumatised asylum seekers and refugees: results of a pilot study. *European Journal of Psychotraumatology;* 2: 5881. |
| 1. Weine S, Kulauzovic Y, Klebic A, Besic S, Mujagic A, Muzurovic J, Spahovic D, Sclove S, Pavkovic I, Feetham S, Rolland J (2008). Evaluating a multiple-family group access intervention for refugees with PTSD. *Journal of Marital and Family Therapy;* 34: 149 –164. |
| 1. Weinstein N, Khabbaz F, Legate N. Enhancing need satisfaction to reduce psychological distress in Syrian refugees (2016). *J Consult Clin Psychol;* 84(7):645-50. |

**EXCLUDED STUDIES**

| REFERENCE OF EXCLUDED STUDIES | REASON |
| --- | --- |
| 1. Ainamani HE, Elbert T, Olema DK, Hecker T (2017). PTSD symptom severity relates to cognitive and psycho-social dysfunctioning–a study with Congolese refugees in Uganda. *European journal of psychotraumatology*; 8(1), 1283086. | Wrong study design |
| 1. Alghamdi M, Hunt N, Thomas S (2015). The effectiveness of Narrative Exposure Therapy with traumatised firefighters in Saudi Arabia: A randomized controlled study. *Behaviour research and therapy*; 66, 64-71. | Wrong population |
| 1. Arntz A, Sofi D, van Breukelen G (2013). Imagery Rescripting as treatment for complicated PTSD in refugees: a multiple baseline case series study. *Behaviour Research and Therapy;* 51(6): 274-83 | Wrong study design |
| 1. Asukai N, Saito A, Tsuruta N, Kishimoto J, Nishikawa T (2010). Efficacy of exposure therapy for Japanese patients with posttraumatic stress disorder due to mixed traumatic events: A randomized controlled study. *Journal of traumatic stress*; *23*(6), 744-750. | Wrong population |
| 1. Bass J, Neugebauer R, Clougherty KF, Verdeli H, Wickramaratne P, Ndogoni L, Speelman L, Weissman M, Bolton P (2006). Group interpersonal psychotherapy for depression in rural Uganda: 6-month outcomes. *The British Journal of Psychiatry*; 188(6), 567-573. | Wrong population |
| 1. Bass J, Murray SM, Mohammed TA, Bunn M, Gorman W, Ahmed AM, Murray L, Bolton P. (2016). A randomized controlled trial of a trauma-informed support, skills, and psychoeducation intervention for survivors of torture and related trauma in Kurdistan, Northern Iraq. *Global Health: Science and Practice*; 4(3), 452-466. | Wrong population |
| 1. Beck BD, Messel C, Meyer SL, Cordtz TO, Søgaard U, Simonsen E, Moe T (2018). Feasibility of trauma-focused Guided Imagery and Music with adult refugees diagnosed with PTSD: A pilot study. *Nordic Journal of Music Therapy;* 27(1), 67–86. | Wrong study design |
| 1. Betancourt TS, Newnham EA, Brennan RT, Verdeli H, Borisova I, Neugebauer R, Bass J, Bolton P (2012). Moderators of treatment effectiveness for war-affected youth with depression in northern Uganda. *Journal of Adolescent Health*; 51(6), 544-550. | Wrong population |
| 1. Bichescu D, Neuner F, Schauer M, Elbert T (2007). Narrative exposure therapy for political imprisonment-related chronic posttraumatic stress disorder and depression. *Behaviour research and therapy*; 45(9), 2212-2220. | Wrong population |
| 1. Birman D, Beehler S, Harris EM, Everson ML, Batia K, Liautaud J, Frazier S, Atkins M, Blanton S, Buwalda J, Fogg L, Cappella E (2008). International Family, Adult, and Child Enhancement Services (FACES): a community-based comprehensive services model for refugee children in resettlement. *American Journal of* *Orthopsychiatry*; 78(1), 121. | Wrong study design |
| 1. Bolton P, Bass J, Neugebauer R, Verdeli H, Clougherty KF, Wickramaratne P, Speelman L, Ndogoni L, Weissman M (2003). Group interpersonal psychotherapy for depression in rural Uganda: a randomized controlled trial. *Jama*; 289(23), 3117-3124. | Wrong population |
| 1. Bolton P, Bass JK, Zangana GA, Kamal T, Murray SM6, Kaysen D, Lejuez CW, Lindgren K, Pagoto S, Murray LK, Van Wyk SS, Ahmed AM, Amin NM, Rosenblum M. (2014). A randomized controlled trial of mental health interventions for survivors of systematic violence in Kurdistan, Northern Iraq. *BMC psychiatry*; 14(1), 360. | Wrong population |
| 1. Böttche M, Kuwert P, Pietrzak RH, Knaevelsrud C (2016). Predictors of outcome of an Internet‐based cognitive‐behavioural therapy for post‐traumatic stress disorder in older adults. *Psychology and Psychotherapy: Theory, Research and Practice*; 89(1), 82-96. | Wrong population |
| 1. Bryant RA, Schafer A, Dawson KS, Anjuri D, Mulili C, Ndogoni L, Koyiet P, Sijbrandij M, Ulate J, Harper Shehadeh M, Hadzi-Pavlovic D, van Ommeren M (2017). Effectiveness of a brief behavioural intervention on psychological distress among women with a history of gender-based violence in urban Kenya: A randomised clinical trial. *PLoS Med;* 14(8):e1002371. | Wrong population |
| 1. Catani C, Kohiladevy M, Ruf M, Schauer E, Elbert T, Neuner F (2009). Treating children traumatized by war and Tsunami: a comparison between exposure therapy and meditation-relaxation in North-East Sri Lanka. *BMC psychiatry*; 9(1), 22. | Wrong population |
| 1. Dajani R, Hadfield K, van Uum S, Greff M, Panter-Brick C (2017). Hair cortisol concentrations in war-affected adolescents: A prospective intervention trial. *Psychoneuroendocrinology;* 89:138-146. | Wrong population |
| 1. Dawson KS, Schafer A, Anjuri D, Ndogoni L, Musyoki C, Sijbrandij M, van Ommeren M, Bryant RA (2016). Feasibility trial of a scalable psychological intervention for women affected by urban adversity and gender-based violence in Nairobi. *BMC Psychiatry;* 16(1):410. | Wrong population |
| 1. Drozdek B (1997). Follow-up study of concentration camp survivors from Bosnia-Herzegovina: three years later. *The Journal of nervous and mental disease*; 185(11), 690-694. | Wrong study design |
| 1. Drožđek B, Bolwerk N (2010). Evaluation of group therapy with traumatized asylum seekers and refugees—The Den Bosch Model. *Traumatology*; 16(4), 117. | Wrong study design |
| 1. Droždek B, Kamperman AM, Bolwerk N, Tol WA, Kleber RJ. (2012). Group therapy with male asylum seekers and refugees with posttraumatic stress disorder: A controlled comparison cohort study of three day-treatment programs. *The Journal of nervous and mental disease*; 200(9), 758-765. | Wrong study design |
| 1. Ertl V, Pfeiffer A, Schauer E, Elbert T, Neuner F (2011). Community-implemented trauma therapy for former child soldiers in Northern Uganda: a randomized controlled trial. *Jama*; 306(5), 503-512. | Wrong population |
| 1. Esala JJ, Taing S (2017). Testimony Therapy With Ritual: A Pilot Randomized Controlled Trial. *Journal of traumatic stress*; 30(1), 94-98. | Wrong population |
| 1. Gordon JS, Staples JK, Blyta A, Bytyqi M, Wilson AT (2008). Treatment of posttraumatic stress disorder in postwar Kosovar adolescents using mind-body skills groups: a randomized controlled trial. *The Journal of clinical psychiatry*; 69(9):1469-76. | Wrong population |
| 1. Halvorsen JØ, Stenmark H (2010). Narrative exposure therapy for posttraumatic stress disorder in tortured refugees: a preliminary uncontrolled trial. S*candinavian Journal of Psychology;* 51(6):495-502. | Wrong study design |
| 1. Hensel-Dittmann D, Schauer M, Ruf M, Catani C, Odenwald M, Elbert T, Neuner F (2011). Treatment of traumatized victims of war and torture: a randomized controlled comparison of narrative exposure therapy and stress inoculation training. *Psychotherapy and Psychosomatics*; 80(6):345–52. | Active comparator |
| 1. Hijazi AM (2012). *Narrative exposure therapy to treat traumatic stress in middle eastern refugees: a clinical trial* (Doctoral dissertation, Wayne State University). | Wrong pubication type |
| 1. Jespersen KV, Vuust P (2012). The effect of relaxation music listening on sleep quality in traumatized refugees: A pilot study. Journal of music therapy, 49(2), 205-229. | Wrong study design |
| 1. Kananian S, Ayoughi S, Farugie A, Hinton D, Stangier U (2017). Transdiagnostic culturally adapted CBT with Farsi-speaking refugees: a pilot study. *Eur J Psychotraumatol;* 8(sup2):1390362. | Wrong study design |
| 1. Kangaslampi S, Garoff F, Peltonen K (2015). Narrative exposure therapy for immigrant children traumatized by war: study protocol for a randomized controlled trial of effectiveness and mechanisms of change. *BMC psychiatry*; 15(1), 127. | Wrong study design |
| 1. Khan MN, Hamdani SU, Chiumento A, Dawson K, Bryant RA, Sijbrandij M, Nazir H, Akhtar P, Masood A, Wang D, Wang E, Uddin I, Ommeren MV, Rahman A (2017). Evaluating feasibility and acceptability of a group WHO trans-diagnostic intervention for women with common mental disorders in rural Pakistan: a cluster randomised controlled feasibility trial. *Epidemiol Psychiatr* Sci; 10:1-11. | Wrong population |
| 1. Knaevelsrud C, Liedl A, Maercker A (2010). Posttraumatic growth, optimism and openness as outcomes of a cognitive-behavioural intervention for posttraumatic stress reactions. *Journal of health psychology;* 15(7):1030-8. | Wrong population |
| 1. Knaevelsrud C, Maercker A (2007). Internet-based treatment for PTSD reduces distress and facilitates the development of a strong therapeutic alliance: a randomized controlled clinical trial. *BMC Psychiatry;* 7, 13. | Wrong population |
| 1. Kruse J, Joksimovic L, Cavka M, Wöller W, Schmitz N (2009). Effects of trauma‐focused psychotherapy upon war refugees. *Journal of Traumatic Stress*; *22*(6), 585-592. | Wrong study design |
| 1. Kulwicki A, Ballout S (2015). Post Traumatic Stress Disorder (PTSD) in Arab American refugee and recent immigrant women. *Journal of Cultural Diversity*, *22*(1). | Wrong intervention |
| 1. McMullen J, O'callaghan P, Shannon C, Black A, Eakin J (2013). Group trauma‐focused cognitive‐behavioural therapy with former child soldiers and other war‐affected boys in the DR Congo: A randomised controlled trial. *Journal of Child Psychology and Psychiatry*; 54(11), 1231-1241. | Wrong population |
| 1. Muller J, Karl A, Denke C, Mathier F, Dittmann J, Rohleder N, Knaevelsrud C (2009). Biofeedback for pain management in traumatised refugees. *Cogn Behav Ther*; 38(3):184-90. | Wrong study design |
| 1. Neuner F, Catani C, Ruf M, Schauer E, Schauer M, Elbert T (2008). Narrative exposure therapy for the treatment of traumatized children and adolescents (KidNET): from neurocognitive theory to field intervention. *Child and adolescent psychiatric clinics of North America*; 17(3), 641-664. | Wrong study design |
| 1. Nordbrandt MS, Carlsson J, Lindberg LG, Sandahl H, Mortensen EL (2015). Treatment of traumatised refugees with basic body awareness therapy versus mixed physical activity as add-on treatment: Study protocol of a randomised controlled trial. *Trials*; 16(1), 477. | Wrong study design |
| 1. Onyut LP, Neuner F, Schauer E, Ertl V, Odenwald M, Schauer M, Elbert T (2004). The Nakivale Camp Mental Health Project: Building local competency for psychological assistance to traumatised refugees. *Intervention*; 2(2), 90-107. | Wrong study design |
| 1. Palic S, Elklit A (2009). An explorative outcome study of CBT-based multidisciplinary treatment in a diverse group of refugees from a Danish treatment centre for rehabilitation of traumatized refugees. *Torture;* 19(3):248-70. | Wrong study design |
| 1. Panter-Brick C, Dajani R, Eggerman M, Hermosilla S, Sancilio A, Ager A (2017). Insecurity, distress and mental health: experimental and randomized controlled trials of a psychosocial intervention for youth affected by the Syrian crisis. *J Child Psychol Psychiatry;* doi: 10.1111/jcpp.12832. | Wrong population |
| 1. Paunovic N, Öst LG (2001). Cognitive-behavior therapy vs. exposure therapy in the treatment of PTSD in refugees. *Behaviour Research and Therapy;* 39: 1183–1197. | Active comparator |
| 1. Quinlan R, Schweitzer RD, Khawaja N, Griffin J (2016). Evaluation of a school-based creative arts therapy program for adolescents from refugee backgrounds. *The Arts in Psychotherapy*; 47, 72-78. | Wrong study design |
| 1. Rahman A, Hamdani SU, Awan NR, Bryant RA, Dawson KS, Khan MF, Azeemi MM, Akhtar P, Nazir H, Chiumento A, Sijbrandij M, Wang D, Farooq S, van Ommeren M (2016). Effect of a Multicomponent Behavioral Intervention in Adults Impaired by Psychological Distress in a Conflict Affected Area of Pakistan: A Randomized Clinical Trial. *JAMA*; 316(24):2609-2617. | Wrong population |
| 1. Rees B, Travis F, Shapiro D, Chant R (2013). Reduction in posttraumatic stress symptoms in Congolese refugees practicing transcendental meditation. *Journal of traumatic stress*; 26(2), 295-298. | Wrong study design |
| 1. Renner W (2009). The effectiveness of psychotherapy with refugees and asylum seekers: preliminary results from an Austrian study. *Journal of Immigrant and Minority Health;* 11(1):41-5. | Wrong study design |
| 1. Rousseau C, Benoit M, Gauthier MF, Lacroix L, Alain N, Rojas MV, Moran A, Bourassa D (2007). Classroom drama therapy program for immigrant and refugee adolescents: A pilot study. *Clinical child psychology and psychiatry*; 12(3), 451-465. | Wrong population |
| 1. Rousseau C, Beauregard C, Daignault K, Petrakos H4, Thombs BD, Steele R, Vasiliadis HM, Hechtman L (2014). A cluster randomized-controlled trial of a classroom-based drama workshop program to improve mental health outcomes among immigrant and refugee youth in special classes. *PloS on;*9*(8*), e104704. | Wrong population |
| 1. Schaal S, Elbert T, Neuner F (2009). Narrative exposure therapy versus interpersonal psychotherapy. *Psychotherapy and psychosomatics*; 78(5), 298-306. | Wrong population |
| 1. Schottelkorb AA, Doumas DM, Garcia R (2012). Treatment for childhood refugee trauma: A randomized, controlled trial. *International Journal of Play Therapy*; 21(2), 57. | Wrong population |
| 1. Smajkić A, Weine S, Durić-Bijedić Z, Boskailo E, Lewis J, Pavković I (2001). Sertralilne, paroxetine and venlafaxine in refugee post traumatic stress disorder with depression symptoms. *Medicinski arhiv*; 55(1 Suppl 1). | Wrong intervention |
| 1. Sonne C, Carlsson J, Bech P, Elklit A, Mortensen EL (2016). Treatment of trauma-affected refugees with venlafaxine versus sertraline combined with psychotherapy-a randomised study. *BMC psychiatry*; 16(1), 383. | Wrong intervention |
| 1. Stein BD, Jaycox LH, Kataoka SH, Wong M, Tu W, Elliott MN, Fink A (2003). A mental health intervention for schoolchildren exposed to violence: A randomized controlled trial. *Jama*; 290(5), 603-611. | Wrong population |
| 1. Steinert C, Bumke PJ, Hollekamp RL, Larisch A, Leichsenring F, Mattheß H, Sek S, Sodemann U, Stingl M, Ret T, Vojtová H, Wöller W, Kruse J (2017). Resource activation for treating post-traumatic stress disorder, co-morbid symptoms and impaired functioning: a randomized controlled trial in Cambodia. *Psychological medicine*; 47(3), 553-564. | Wrong population |
| 1. Stenmark H, Catani C, Elbert T, Gøtestam KG (2008). Narrative Exposure Therapy compared to treatment as usual for refugees with PTSD-Preliminary results from a randomized controlled trial. *European Psychiatry*; 23, S90. | Wrong study design |
| 1. Vijayakumar L, Mohanraj R, Kumar S, Jeyaseelan V, Sriram S, Shanmugam M (2017). CASP - An intervention by community volunteers to reduce suicidal behaviour among refugees. *Int J Soc Psychiatry;* 63(7):589-597. | Wrong study design |
| 1. Weiss WM, Murray LK, Zangana GA, Mahmooth Z, Kaysen D, Dorsey S, Lindgren K, Gross A, Murray SM, Bass JK, Bolton P (2015). Community-based mental health treatments for survivors of torture and militant attacks in Southern Iraq: a randomized control trial. *BMC psychiatry*; 15(1), 249. | Wrong population |
| 1. Yeomans PD, Forman EM, Herbert JD, Yuen E (2010). A randomized trial of a reconciliation workshop with and without PTSD psychoeducation in Burundian sample. *Journal of traumatic stress*; 23(3), 305-312. | Wrong population |

**AWAITING ASSESSMENT**

| REFERENCES TO STUDIES AWAITING ASSESSMENT |
| --- |
| 1. Nguvu: Evaluating an integrated approach to reduce intimate partner violence and psychological distress in refugees in Tanzania (ISRCTN65771265). |
| 1. Mental Health in Refugees and Asylum Seekers (MEHIRA) (NCT03109028). |
| 1. A Psychosocial Program Impact Evaluation in Jordan (NCT03012451). |

**RISK OF BIAS GRAPH**

Review authors' judgements about each risk of bias item presented as percentages across all included studies.


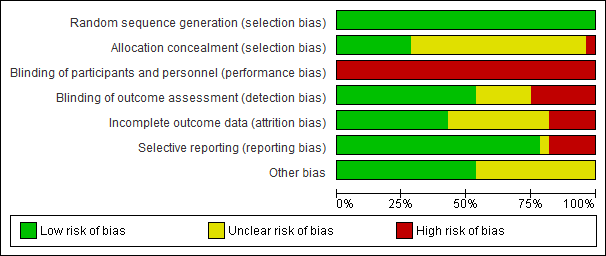


**RISK OF BIAS SUMMARY**

Review authors' judgements about each risk of bias item for each included study.


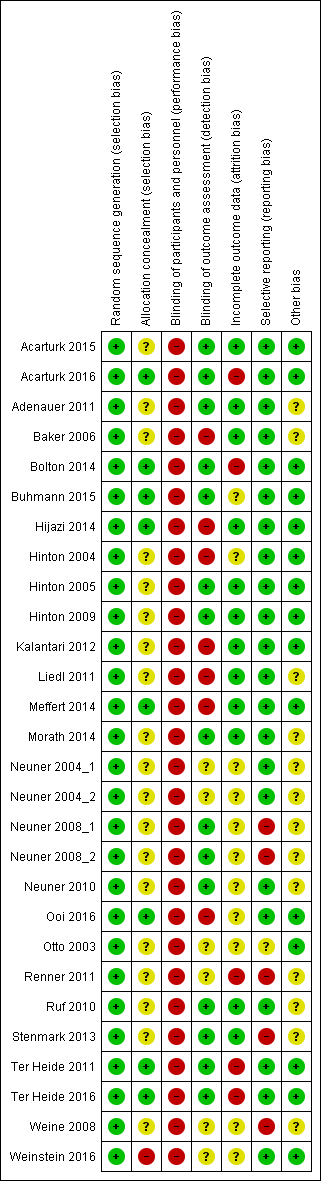


**RISK OF BIAS: REVIEW AUTHORS’ JUDGMENTS ABOUT EACH RISK OF BIAS ITEM FOR EACH INCLUDED STUDY**

Acarturk 2015

| **Bias** | **Authors' judgement** | **Support for judgement** |
| --- | --- | --- |
| Random sequence generation (selection bias) | Low risk | QUOTE: "The selection was conducted by using a computer-generated random number list. [...] Participants were randomly assigned on a 1:1 basis to the EMDR or wait-list group". |
| Allocation concealment (selection bias) | Unclear risk | No information provided. |
| Blinding of participants and personnel (performance bias) | High risk | Because of the type of treatment, quote: "The participants and the therapists were aware of the allocated arm". |
| Blinding of outcome assessment (detection bias) | Low risk | QUOTE: "[...] the outcome assessors were kept blind to the allocation". |
| Incomplete outcome data (attrition bias) | Low risk | All randomised patients completed the study and there were no missing data. Results were reported for all randomised patients. |
| Selective reporting (reporting bias) | Low risk | Protocol is not available, however all expected outcomes were clearly reported at post-treatment and follow-up. |
| Other bias | Low risk | All participants were Syrian and all interviews were carried out in the local language, with the help of Syrian interpreters. The measures were translated into Arabic. Sponsorship bias is unlikely to have occurred. |

Acarturk 2016

| **Bias** | **Authors' judgement** | **Support for judgement** |
| --- | --- | --- |
| Random sequence generation (selection bias) | Low risk | QUOTE: "Participants were randomly assigned on a 1:1 basis to the EMDR or wait-list group." |
| Allocation concealment (selection bias) | Low risk | QUOTE: "After including the participants, another researcher, not involved in the current study, used a computer-generated random-number list for the allocation of participants to different treatment groups." |
| Blinding of participants and personnel (performance bias) | High risk | QUOTE: "The participants and the therapists were necessarily aware of the allocated arm" because of the type of treatment. |
| Blinding of outcome assessment (detection bias) | Low risk | QUOTE: "[...] the outcome assessors were kept blind to the allocation". |
| Incomplete outcome data (attrition bias) | High risk | Dropouts were high in the two groups: 18/49 (36.73%) in EMDR group and 16/49 (32.65%) in WL group. An intent-to-treat was performed, quote: "provide a robust test of the efficacy of the treatment, and to follow the intention-to-treat principles of data analysis, the missing data points in the ÷2 analyses were replaced with values that would indicate that drop-outs retained the diagnosis of trauma after the intervention." Analysis were apparently performed on all randomized patients and authors stated that the completers’ analyses of the measures produced the same results. |
| Selective reporting (reporting bias) | Low risk | All outcomes were clearly pre-specified in the protocol and were well reported at post-treatment and follow-up. |
| Other bias | Low risk | All participants were Syrian and all interviews were carried out in the local language, with the help of Syrian interpreters. The measures were translated into Arabic. Sponsorship bias is unlikely to have occurred. |

Adenauer 2011

| **Bias** | **Authors' judgement** | **Support for judgement** |
| --- | --- | --- |
| Random sequence generation (selection bias) | Low risk | QUOTE: "participants... were randomised using a computer-generated list of random numbers". |
| Allocation concealment (selection bias) | Unclear risk | No information provided. |
| Blinding of participants and personnel (performance bias) | High risk | Participants were not blind (waiting list); personnel cannot be blind for this type of treatment. |
| Blinding of outcome assessment (detection bias) | Low risk | Posttest were carried out by interviewers who were blind to treatment condition. |
| Incomplete outcome data (attrition bias) | Low risk | Analysis carried out only on completers; however, the number of dropout is very low, balanced across intervention groups with similar reasons for missing data across groups, that are not related to the outcome (patients moved for deportation). |
| Selective reporting (reporting bias) | Low risk | All outcomes were clearly pre-specified in the protocol and were clearly reported in the paper. |
| Other bias | Unclear risk | NET was carried out with the help of interpreters if necessary. Two of the authors of the paper are authors of the NET manual. |

Baker 2006

| **Bias** | **Authors' judgement** | **Support for judgement** |
| --- | --- | --- |
| Random sequence generation (selection bias) | Low risk | QUOTE: "students from each separate class group were randomly allocated into Group 1 or Group 2 to obtain balance across age groups and academic level". No information about the sequence generation process. |
| Allocation concealment (selection bias) | Unclear risk | No information provided. |
| Blinding of participants and personnel (performance bias) | High risk | Participants and therapists were not blind, because of the study design. |
| Blinding of outcome assessment (detection bias) | High risk | Students were evaluated by their classroom teachers. |
| Incomplete outcome data (attrition bias) | Low risk | There were no missing data. |
| Selective reporting (reporting bias) | Low risk | Protocol is not available, however all expected outcomes were clearly reported. |
| Other bias | Unclear risk | Music therapy was carried out in english, however only few activities implied language skills. No information provided about the sponsorship. |

Bolton 2014

| **Bias** | **Authors' judgement** | **Support for judgement** |
| --- | --- | --- |
| Random sequence generation (selection bias) | Low risk | QUOTE: "The project site director generated these random numbers using STATA. Counselors opened a pre-sealed envelope (corresponding to the ID number) containing assignment to immediate treatment or wait-list." |
| Allocation concealment (selection bias) | Low risk | See above. |
| Blinding of participants and personnel (performance bias) | High risk | This is a single-blinded study: non-blinding of service providers and participants. |
| Blinding of outcome assessment (detection bias) | Low risk | QUOTE: "interviewers at baseline and follow-up did not know to which study arm the interviewees belonged." |
| Incomplete outcome data (attrition bias) | High risk | An ITT analysis was performed, QUOTE: "Missing data, including follow-up scores for those lost to follow-up, were imputed using STATA’s chained equations command for multiple imputation, which pools data according to Rubin’s rules. Baseline and follow-up scores on all items missing data were then imputed using all of the variables in the dataset. All analyses used the full intent-to-treat (ITT) sample."  However, drop-out were high in the control group (23.6%; 39/165), and authors stated that "It is quite likely that the missing data have affected our outcome estimates, including the effect sizes, although the size and direction of the change is not known". |
| Selective reporting (reporting bias) | Low risk | All outcomes were clearly pre-specified in the protocol and were clearly reported. |
| Other bias | Low risk | QUOTE: "Funding was provided by the USAID Victims of Torture Fund. No funding bodies had any role in study design, data collection and analysis, decision to publish, or preparation of the manuscript."  Sponsorship bias is unlikely to have occurred. |

Buhmann 2015

| **Bias** | **Authors' judgement** | **Support for judgement** |
| --- | --- | --- |
| Random sequence generation (selection bias) | Low risk | QUOTE: "the randomisation sequence was computer generated by the Department of Biostatistics at University of Copenhagen, which was not otherwise involved in the research project. Randomisation was stratified by gender and total score on HTQ, so that patients with equal illness severity were allocated to all groups." |
| Allocation concealment (selection bias) | Low risk | QUOTE: "allocation was concealed by using sequentially numbered sealed envelopes. The envelopes were kept in an office physically separate from the clinic and were administered by secretaries who were not associated with the research project. When a patient had been included in the trial, the physician telephoned the office administering the randomisation envelopes and patients were subsequently assigned to a treatment group." |
| Blinding of participants and personnel (performance bias) | High risk | QUOTE: "it was not deemed possible to mask the patients, physicians, or psychologists to the treatment group because of the substantial differences between the treatment modalities". |
| Blinding of outcome assessment (detection bias) | Low risk | QUOTE: "a group of medical students not otherwise involved in the treatment undertook the masked ratings and met regularly to increase rater reliability" |
| Incomplete outcome data (attrition bias) | Unclear risk | Missing data have been imputed using appropriated method (full information maximum likelihood- FIML); however number of patients included in the analyses corresponds to completers only. Data on drop-out rates are unclear. |
| Selective reporting (reporting bias) | Low risk | All expected outcomes were clearly Pre-specified in the protocol and well reported in the paper. |
| Other bias | Low risk | QUOTE: "All self-report questionnaires were available in the six most common languages at the clinic, which included the languages of 92% of patients. If no translation was available, an interpreter translated the official version into the language of the patient."  The trial was funded by the capital region of Copenhagen.  Sponsorship bias is unlikely to have occurred. |

Hijazi 2014

| **Bias** | **Authors' judgement** | **Support for judgement** |
| --- | --- | --- |
| Random sequence generation (selection bias) | Low risk | QUOTE: "the computerized scheme was stratified by recruitment site (agency) and assistance, and randomised the two conditions in blocks of six in a 2:1 ratio". |
| Allocation concealment (selection bias) | Low risk | QUOTE: "the assistant (heretofore blind to condition assignment) opened a sealed envelope and informed the participants when he or she would be getting the treatment". |
| Blinding of participants and personnel (performance bias) | High risk | Participants and personnel not blind (see above). |
| Blinding of outcome assessment (detection bias) | High risk | Measures were self-administered by the patients. QUOTE: "all participants were mailed follow up assessment measure and returned envelopes 2 and 4 months after measure". |
| Incomplete outcome data (attrition bias) | Low risk | QUOTE: "our primary analyses were intent-to-treat, meaning that we retained all 36 participants, regardless of how many intervention or follow-up assessment sessions they completed. Any missing follow-up data were replaced using the multiple imputation procedure in SPSS." |
| Selective reporting (reporting bias) | Low risk | All expected outcomes were clearly pre-specified in the protocol and well reported in the paper at all follow-up. |
| Other bias | Low risk | The personnel were Arabic-speaking as the participants and the measures were translated into Arabic and most of the translated versions were validated. This research was supported by the Blue Cross Blue Shield of Michigan Foundation and award RO1 057808 from the National Institute of Arthritis,Musculoskeletal, and Skin Diseases. Sponsorship bias is unlikely to have occurred. |

Hinton 2004

| **Bias** | **Authors' judgement** | **Support for judgement** |
| --- | --- | --- |
| Random sequence generation (selection bias) | Low risk | QUOTE: "the patients were randomly assigned to two cohorts of 6 each". No further details provided. |
| Allocation concealment (selection bias) | Unclear risk | No information provided. |
| Blinding of participants and personnel (performance bias) | High risk | Participants not blind (waiting list); personnel cannot be blind for this type of treatment. |
| Blinding of outcome assessment (detection bias) | High risk | Measures were self-administered by the patients. QUOTE: "the participants completed the measures at three time points". |
| Incomplete outcome data (attrition bias) | Unclear risk | Data are provided for all randomised patients. No details provided on drop-out and eventual methods to impute missing data |
| Selective reporting (reporting bias) | Low risk | Protocol is not available, however all expected outcomes were clearly reported at all follow-up. |
| Other bias | Low risk | One therapist led the CBT session and Vietnamese social workers and staff provided translation and cultural consultation; all patients were Vietnamese; the measures were translated and validated for Vietnamese population. Sponsorship bias is unlikely to have occurred. |

Hinton 2005

| **Bias** | **Authors' judgement** | **Support for judgement** |
| --- | --- | --- |
| Random sequence generation (selection bias) | Low risk | QUOTE: "patients... were stratified by gender, with random allocation to either the Initial treatment, or the Delayed Treatment Groups decided by a coin toss". |
| Allocation concealment (selection bias) | Unclear risk | No information provided. |
| Blinding of participants and personnel (performance bias) | High risk | Participants not blind (waiting list); personnel cannot be blind for this type of treatment. |
| Blinding of outcome assessment (detection bias) | Low risk | QUOTE: "blind to treatment condition, all assessments were made by a Cambodian bicultural worker". |
| Incomplete outcome data (attrition bias) | Low risk | Data are provided for all randomised patients; all randomised patients completed the study and there were no missing data. |
| Selective reporting (reporting bias) | Low risk | Protocol is not available, however all expected outcomes were clearly reported at all follow-up. |
| Other bias | Low risk | All patients were Cambodian and CBT session were conducted by one therapist who is fluent in Cambodian; all measures were translated and then back-translated. Sponsorship bias is unlikely to have occurred. |

Hinton 2009

| **Bias** | **Authors' judgement** | **Support for judgement** |
| --- | --- | --- |
| Random sequence generation (selection bias) | Low risk | QUOTE: "Eligible patients who agreed to participate were stratified by gender, with random allocation to either initial or delayed treatment decided by a coin toss." |
| Allocation concealment (selection bias) | Unclear risk | No information provided. |
| Blinding of participants and personnel (performance bias) | High risk | Personnel and participants cannot be blind for this type of treatment. |
| Blinding of outcome assessment (detection bias) | Low risk | QUOTE: "Blind to treatment condition, all assessments were made by a Cambodian bicultural worker". |
| Incomplete outcome data (attrition bias) | Low risk | Data are provided for all randomised patients; all randomised patients completed the study and there were no missing data. |
| Selective reporting (reporting bias) | Low risk | Protocol is not available. All expected outcomes were clearly reported at all time points. |
| Other bias | Low risk | The first author, who is fluent in Cambodian, conducted or co-led the intervention and all measures were translated and then back-translated.  No information provided about the sponsorship, however sponsorship bias is unlikely to have occurred. |

Kalantari 2012

| **Bias** | **Authors' judgement** | **Support for judgement** |
| --- | --- | --- |
| Random sequence generation (selection bias) | Low risk | QUOTE: "64 were randomly assigned to either experimental (n = 32) or control groups (n = 32)". No information provided about the sequence generation process. |
| Allocation concealment (selection bias) | Unclear risk | No information provided. |
| Blinding of participants and personnel (performance bias) | High risk | Participants cannot be blind for this type of treatment and no further information was provided about the blinding of personnel. |
| Blinding of outcome assessment (detection bias) | High risk | Questionnaire was self-administered by the participants, who were not blind to the allocation. |
| Incomplete outcome data (attrition bias) | Low risk | Drop-out rates were low (3/32, 9.37% in the experimental group and 0% in the control group) and data were reported only for completers. |
| Selective reporting (reporting bias) | Low risk | Protocol is not available. Data are well reported in the paper. |
| Other bias | Low risk | A Farsi version of TGIC was developed for use in this study.  This study was based on a grant awarded from Children and War Foundation. Sponsorship bias is unlikely to have occurred. |

Liedl 2011

| **Bias** | **Authors' judgement** | **Support for judgement** |
| --- | --- | --- |
| Random sequence generation (selection bias) | Low risk | QUOTE: "...were randomly assigned to one of the three conditions". |
| Allocation concealment (selection bias) | Unclear risk | No information provided. |
| Blinding of participants and personnel (performance bias) | High risk | Participants not blind (waiting list); personnel cannot be blind for this type of treatment. |
| Blinding of outcome assessment (detection bias) | High risk | QUOTE: "The questionnaire were administered using multilingual computer assisted self-interview..." |
| Incomplete outcome data (attrition bias) | Low risk | Only completers data were analysed; missing outcome data were low and balanced in numbers across intervention groups, with similar reasons for missing data across groups. |
| Selective reporting (reporting bias) | Low risk | Protocol is not available. All expected outcomes were clearly reported. |
| Other bias | Unclear risk | QUOTE: "wherever possible, we used validated version of the questionnaire in the participants native languages". Paper was retracted. |

Meffert 2014

| **Bias** | **Authors' judgement** | **Support for judgement** |
| --- | --- | --- |
| Random sequence generation (selection bias) | Low risk | QUOTE: "Participants were randomly assigned IPT or the waitlist. Pure randomization was used with a random allocation sequence." |
| Allocation concealment (selection bias) | Low risk | QUOTE: "Eligible participants were randomly assigned to IPT or waitlist control groups using a computer-generated random allocation sequence." |
| Blinding of participants and personnel (performance bias) | High risk | QUOTE: "Participants were not blinded to group status [...] Therapists were not blind to group status [...] given the nature of the intervention". |
| Blinding of outcome assessment (detection bias) | High risk | The administrators of the measurements were the future (or former) therapists of the participants who were not blind to group status. |
| Incomplete outcome data (attrition bias) | Low risk | Drop-out rates was low (2/13, 15.3% in the experimental group and 1/9, 11% in the control group) and data were reported for completers. However, authors stated that, quote: "To address the effects of missing data, dropouts, and the one case lost to follow-up, we completed a last observation carried forward (LOCF) analysis.[...] these findings were not substantially changed by an intent-to-treat analysis". |
| Selective reporting (reporting bias) | Low risk | All expected outcomes were clearly reported. |
| Other bias | Low risk | The instruments were translated using a standardized method of instrument adaptation and translation, as described by the World Health Organization. Community members were trained to deliver the care. This work was supported by a University of California, San Francisco Academic Senate Research Grant and a sponsorship bias is unlikely to have occurred. |

Morath 2014

| **Bias** | **Authors' judgement** | **Support for judgement** |
| --- | --- | --- |
| Random sequence generation (selection bias) | Low risk | QUOTE: "An independent person randomly assigned individuals with PTSD to either a treatment condition (NET) or the WLC group using permuted blocks of variable length". |
| Allocation concealment (selection bias) | Unclear risk | No information provided. |
| Blinding of participants and personnel (performance bias) | High risk | Participants not blind (waiting list); personnel cannot be blind for this type of treatment. |
| Blinding of outcome assessment (detection bias) | Low risk | QUOTE: "Diagnosticians were not aware of which participants were allocated to which group. Blinded diagnosticians conducted post-test and follow-up interviews". |
| Incomplete outcome data (attrition bias) | Low risk | QUOTE: Supplement "with respect to missing values, analyses were done using mixed models procedure"; Drop-out were balanced in two groups |
| Selective reporting (reporting bias) | Low risk | All expected pre-specified outcomes were clearly reported at post-test and at follow-up. |
| Other bias | Unclear risk | QUOTE: "Therapists relied on trained interpreters if necessary. Two of the authors of the paper are authors of the NET manual Study was funded by the European Refugee Fund for financial support. |

Neuner 2004

| **Bias** | **Authors' judgement** | **Support for judgement** |
| --- | --- | --- |
| Random sequence generation (selection bias) | Low risk | QUOTE: "Each participant was randomly assigned (using a dice) to one of three treatment groups: narrative exposure therapy, supportive counseling, or psychoeducation only". |
| Allocation concealment (selection bias) | Unclear risk | No information provided. |
| Blinding of participants and personnel (performance bias) | High risk | Personnel cannot be blind for this type of treatment. No information was provided about blinding of participants. |
| Blinding of outcome assessment (detection bias) | Unclear risk | QUOTE: "The local and expert interviewers who carried out the posttests, as well as the follow-up tests, were blind for the individual participant’s treatment condition. The respondents were instructed not to inform the interviewers or the trained researchers about the type of treatment or the number of sessions they had received" but some measures were self-administered by the patients (SRQ-20 e SF-12). |
| Incomplete outcome data (attrition bias) | Unclear risk | QUOTE: "To maximize use of information in this study with a small sample size, missing data were estimated with a restricted maximum likelihood procedure" ... "All participants were included in analyses." |
| Selective reporting (reporting bias) | Low risk | Protocol is not available, however all expected outcomes were clearly reported at post-test and at follow-up. |
| Other bias | Unclear risk | QUOTE: "Self-report instruments were translated into the Arabic dialect spoken by the refugees in Imvepi (Juba-Arabic)"  The authors of the paper are authors of the NET manual;  Research was funded by the Deutsche Forschungsgemeinschaft. |

Neuner 2008

| **Bias** | **Authors' judgement** | **Support for judgement** |
| --- | --- | --- |
| Random sequence generation (selection bias) | Low risk | QUOTE: "The list of participants was ordered randomly; the first 4 were consecutively assigned to the NET, TC, NET, and TC groups; and the fifth was assigned to the MG (monitoring) group. This procedure was repeated until all 277 participants were assigned." |
| Allocation concealment (selection bias) | Unclear risk | No information provided. |
| Blinding of participants and personnel (performance bias) | High risk | Personnel cannot be blind for this type of treatment. Participants of MG were aware about their treatment allocation but the participants who received treatment were blind with respect to the particular treatment condition. |
| Blinding of outcome assessment (detection bias) | Low risk | Interviewers were blind with respect to the particular treatment condition. |
| Incomplete outcome data (attrition bias) | Unclear risk | More than 20% of patients abandoned the study prematurely. Study endpoint: 50/111 missing from NET group; 52/111 missing from TC group.  QUOTE: "Aiming at an intention-to-treat analysis, we included in the outcome analysis all participants who were randomized......we chose to apply mixed-effects models that allow the inclusion of all available data without the arbitrary replacement or imputation of missing values" . |
| Selective reporting (reporting bias) | High risk | Protocol is not available. Only one outcome measure tested. |
| Other bias | Unclear risk | The authors of the paper are authors of the NET manual. Sponsorship bias cannot be ruled out. |

Neuner 2010

| **Bias** | **Authors' judgement** | **Support for judgement** |
| --- | --- | --- |
| Random sequence generation (selection bias) | Low risk | QUOTE: "participants were randomised into the two groups using a block permutation procedure with blocks of four patients". |
| Allocation concealment (selection bias) | Unclear risk | No information provided. |
| Blinding of participants and personnel (performance bias) | High risk | Personnel cannot be blind for this type of treatment. Participants in the control group "received the treatment, including psychotherapy and psychoactive medication. None of the patient in the TAU group received trauma focused treatment". |
| Blinding of outcome assessment (detection bias) | Low risk | QUOTE: "we aimed at keeping interviewers blind to each participant’s condition. However, occasionally, the participants revealed their condition to the interviewer, despite instruction not to do so". |
| Incomplete outcome data (attrition bias) | Unclear risk | QUOTE: "we chose to apply mixed effects models that allow the inclusion all available data....". However results are reported at post treatment only for completers. Only two patients dropped out from the NET group, one for reasons related to the treatment. |
| Selective reporting (reporting bias) | Low risk | Protocol is not available, however all expected outcomes were clearly reported at endpoint. |
| Other bias | Unclear risk | patients were heterogeneous in terms of country of origin.  QUOTE: "All instruments were assessed in the form of structured interviews. NET treatment was carried out according to the manual by therapists from the University of Konstanz with the help of trained interpreters".  Two of the authors of the paper are authors of the NET manual; Study was funded by European Refugee Fund. |

Ooi 2016

| **Bias** | **Authors' judgement** | **Support for judgement** |
| --- | --- | --- |
| Random sequence generation (selection bias) | Low risk | QUOTE: "Each school in a pair was randomly allocated into either the intervention or WL control condition using a computer generated random number by the statistical supervisor of this study (RK) who was not at all involved in the clinical aspects of this study." |
| Allocation concealment (selection bias) | Low risk | See above. |
| Blinding of participants and personnel (performance bias) | High risk | Personnel and participants cannot be blind given the type of treatment. |
| Blinding of outcome assessment (detection bias) | High risk | Assessors were not blind to the treatment condition. |
| Incomplete outcome data (attrition bias) | Unclear risk | Only one participant dropped-out from the study in the intervention group at post-test and no participants from the control group. |
| Selective reporting (reporting bias) | Low risk | All prespecified outcomes were correctly reported, even though sometimes the sample size remain unclear across measures. |
| Other bias | Low risk | The funders had no role in the study design, data collection and analysis, decision to publish, or preparation of the manuscript. Sponsorship bias cannot be ruled out. |

Otto 2003

| **Bias** | **Authors' judgement** | **Support for judgement** |
| --- | --- | --- |
| Random sequence generation (selection bias) | Low risk | QUOTE: "five patients were randomly assigned to sertraline treatment, and five to sertraline treatment plus ten sessions of CBT". No information provided about the sequence generation process. |
| Allocation concealment (selection bias) | Unclear risk | No information provided. |
| Blinding of participants and personnel (performance bias) | High risk | Participants not blind; personnel cannot be blind for this type of treatment. |
| Blinding of outcome assessment (detection bias) | Unclear risk | No information provided. |
| Incomplete outcome data (attrition bias) | Unclear risk | Drop-out data are not reported. |
| Selective reporting (reporting bias) | Unclear risk | Protocol is not available. All expected outcomes were reported, even if the total score of primary outcome is not reported. |
| Other bias | Low risk | All participants were Cambodian (Khmer-speaking); treatment services were provided in Khmer; most of the scales have been validated for Khmer population. |

Renner 2011

| **Bias** | **Authors' judgement** | **Support for judgement** |
| --- | --- | --- |
| Random sequence generation (selection bias) | Low risk | QUOTE: "participants were assigned to the above mentioned conditions at random". Number of patients randomised for each group is not reported. |
| Allocation concealment (selection bias) | Unclear risk | No information provided. |
| Blinding of participants and personnel (performance bias) | High risk | participants not blind (waiting list). Personnel cannot be blind for this type of treatment. |
| Blinding of outcome assessment (detection bias) | Unclear risk | No information provided. |
| Incomplete outcome data (attrition bias) | High risk | QUOTE: "in all the groups there was a substantial drop out rate". Number of drop out for each group is not reported. |
| Selective reporting (reporting bias) | High risk | Protocol is not available and data are reported only for CROP and wait list. Data for CBT and EMDR groups are reported only in graphs. |
| Other bias | Unclear risk | QUOTE: "all the instruments were administered in their written Russian version by the first author after being translated and back translated by professional interpreters. All participants were able to read and understand the questionnaires".  Other bias cannot be ruled out. |

Ruf 2010

| **Bias** | **Authors' judgement** | **Support for judgement** |
| --- | --- | --- |
| Random sequence generation (selection bias) | Low risk | QUOTE: "Children were randomly assigned to either the KIDNET-Group (n = 13) or a wait list control group (n =13)......Permuted blocking randomization was used to generate two groups of similar size for final comparison." |
| Allocation concealment (selection bias) | Unclear risk | No information provided. |
| Blinding of participants and personnel (performance bias) | High risk | participants not blind (waiting list); personnel cannot be blind for this type of treatment. |
| Blinding of outcome assessment (detection bias) | Low risk | QUOTE: "A group of 15 trained clinical psychologists and researchers....administered all instruments and cognitive tests in individual diagnostic interviews. These assessors were left blind about the group assignment". |
| Incomplete outcome data (attrition bias) | Low risk | QUOTE: "As intent-to-treat and treatment-completer analyses revealed the same result, only the treatment-completer analysis will be presented here." Only one dropout in treatment group. |
| Selective reporting (reporting bias) | Low risk | Even if the protocol is not available, all expected outcomes were clearly reported at post-test and at follow-up. |
| Other bias | Unclear risk | QUOTE: "...the interviews were conducted with the help of professional translators who had previously been trained on the concept of PTSD and other mental health disorders".  The authors of the paper are authors of the NET manual;  Research was supported by the European Refugee Fund and the DFG. |

Stenmark 2013

| **Bias** | **Authors' judgement** | **Support for judgement** |
| --- | --- | --- |
| Random sequence generation (selection bias) | Low risk | QUOTE: "Participants were randomized to the treatment conditions by drawing ball from a bag with an a-priori 2/3 chance of receiving NET and 1/3 chance of receiving TAU". |
| Allocation concealment (selection bias) | Unclear risk | No information provided. |
| Blinding of participants and personnel (performance bias) | High risk | Participants not blind and personnel cannot be blind for this type of treatment. |
| Blinding of outcome assessment (detection bias) | Low risk | Single blind: outcomes assessor. QUOTE: "assessor had no access to information about what therapy the patients' had been assigned to and the therapists were instructed not to reveal the type of treatment their patients were given. The aim was to make the assessor as blind as possible to the patients' treatments." |
| Incomplete outcome data (attrition bias) | Low risk | Authors state that intention-to-treat analyses were conducted and that results did not differ from treatment completers. However only completers results are reported. Drop-out were balanced in two groups. |
| Selective reporting (reporting bias) | High risk | Protocol is available and all prespecified outcomes were reported however data are reported only in graphs. |
| Other bias | Unclear risk | There are differences in the background training of the therapists. Patients were heterogeneous in terms of country of origin. QUOTE: "assessment tools were not validated to the language and culture of each participant". Two of the authors of the paper are authors of the NET manual. |

Ter Heide 2011

| **Bias** | **Authors' judgement** | **Support for judgement** |
| --- | --- | --- |
| Random sequence generation (selection bias) | Low risk | QUOTE: "Participants were assigned to their experimental group using simple randomisation through flipping a coin". |
| Allocation concealment (selection bias) | Low risk | QUOTE: "An independent research associate performed randomisation". |
| Blinding of participants and personnel (performance bias) | High risk | Personnel cannot be blind because of the type of treatment. No information about participants. |
| Blinding of outcome assessment (detection bias) | Low risk | QUOTE: "The interview was administered in Dutch by trained, blind assessors. Blindness was maintained in 33 out of 44 assessments (70%)". |
| Incomplete outcome data (attrition bias) | High risk | Drop-out were high in both arms (50%) with similar reasons across groups. Authors stated that no significant differences were found between completers and drop-outs. Primary and secondary outcomes were provided for completers only. |
| Selective reporting (reporting bias) | Low risk | Protocol is not available, however all outcome were reported in the paper. |
| Other bias | Low risk | QUOTE: "Self-report questionnaires were administered in the patient’s native language if possible; interpreters were used when necessary [...] This study was partially funded by ZonMW, the Netherlands organisation for health research and development". Patients were heterogeneous in terms of country of origin. |

Ter Heide 2016

| **Bias** | **Authors' judgement** | **Support for judgement** |
| --- | --- | --- |
| Random sequence generation (selection bias) | Low risk | QUOTE: "A two-arm design was used in which participants were randomly assigned to either 12 h (9 session) of EMDR therapy or 12h (12 sessions) of stabilisation as usual. [...] Participants were assigned to their experimental group through flipping a coin". |
| Allocation concealment (selection bias) | Low risk | QUOTE: "An indipendet research associated who was not otherwise involved in the inclusion process performed randomization." |
| Blinding of participants and personnel (performance bias) | High risk | Personnel cannot be blind because of the type of treatment. No information about participants. |
| Blinding of outcome assessment (detection bias) | Low risk | QUOTE: "Interviews were administered by trained Master’s students in psychology who were kept masked to treatment condition by having limited access to participant data and by asking participants not to reveal treatment content." |
| Incomplete outcome data (attrition bias) | High risk | Drop-out were high in both arms (32.4% in EMDR group and 37.8% in stabilisation group) with similar reasons across groups.  Authors stated that an intent-to-treat analyses for primary outcomes was performed (tab 3): quote "Bayesian analysis enables full intent-to-treat analysis as missing data are automatically imputed." |
| Selective reporting (reporting bias) | Low risk | Protocol is not available, however all outcome were reported in the paper. |
| Other bias | Low risk | QUOTE: "Interpreters were used whenever the participant did not speak Dutch and the instrument was not available in the participant’s native language. This study was jointly funded by ZonMW, The Netherlands organization for health research and development, and Foundation Centrum ’45 [...]".  Patients were heterogeneous in terms of country of origin. |

Weine 2008

| **Bias** | **Authors' judgement** | **Support for judgement** |
| --- | --- | --- |
| Random sequence generation (selection bias) | Low risk | QUOTE: "subjects were randomly assigned to one of the two conditions". No information provided about the sequence generation process. |
| Allocation concealment (selection bias) | Unclear risk | No information provided. |
| Blinding of participants and personnel (performance bias) | High risk | Participants not blind and personnel cannot be blind for this type of treatment. |
| Blinding of outcome assessment (detection bias) | Unclear risk | No details are provided on how the outcome were assessed. |
| Incomplete outcome data (attrition bias) | Unclear risk | No information provided. |
| Selective reporting (reporting bias) | High risk | Protocol is not available. Most of the outcomes were not reported as raw data but only as random effects model. |
| Other bias | Unclear risk | All participants were Bosnian. QUOTE: "all instruments were translate into Bosnian by the research team. Back translations were used to improve the word selection and to verify that questions were understandable to the refugees." The work was supported by the National Institute of Mental Health. |

Weinstein 2016

| **Bias** | **Authors' judgement** | **Support for judgement** |
| --- | --- | --- |
| Random sequence generation (selection bias) | Low risk | QUOTE: "Participants were randomly assigned to one of two conditions: need satisfaction intervention (n 24) or control condition (n 17)." No information provided about the sequence generation process. |
| Allocation concealment (selection bias) | High risk | No information provided. Moreover, authors did not explain because the two groups were unbalanced (n= 24 and n=17). |
| Blinding of participants and personnel (performance bias) | High risk | Participants not blind and the staff cannot be blind for this type of treatment. |
| Blinding of outcome assessment (detection bias) | Unclear risk | No information provided. |
| Incomplete outcome data (attrition bias) | Unclear risk | No information provided about dropout. |
| Selective reporting (reporting bias) | Low risk | Protocol is not available, however all outcome were reported in the paper. |
| Other bias | Low risk | All participants were Syrian and all measures were translated to Arabic and back-translated by an independent experimenter.  No information about sponsorship, however sponsorship bias is unlikely to have occurred. |

**SUBGROUP ANALYSES**

**Subgroup analyses of PTSD outcome (post-treatment)**

| **Meta-analysis** | **Comparisons (N)** | **Patients (N)** | **SMD*** | **95% CI** | ***I*²** | **95% CI** | ***P*** |
| --- | --- | --- | --- | --- | --- | --- | --- |
| Overall PTSD outcomes |  |  |  |  |  |  |  |
| All studies | 21 | 1370 | -0.71 | -1.01 to -0.41 | 83% | 78 to 88 |  |
| **Subgroup analyses** |  |  |  |  |  |  |  |
| **Country** |  |  |  |  |  |  | 0.420 |
| High income | 12 | 514 | -0.59 | -0.97 to -0.21 | 73% | 52 to 85 |  |
| Upper, lower middle, low income | 8 | 856 | -0.84 | -1.33 to -0.35 | 89% | 81 to 93 |  |
| **Control condition** |  |  |  |  |  |  | < 0.001 |
| TAU or no treatment | 7 | 249 | -0.92 | -1.50 to -0.34 | 76% | 49 to 88 |  |
| WL | 10 | 807 | -0.81 | -1.27 to -0.36 | 86% | 76 to 92 |  |
| Psychological placebo | 4 | 314 | -0.02 | -0.25 to 0.20 | 0% | 0 to 85 |  |
| **Age** |  |  |  |  |  |  | 0.250 |
| Adult | 17 | 1158 | -0.78 | -1.15 to -0.42 | 85% | 78 to 90 |  |
| Children and mixed | 4 | 212 | -0.45 | -0.88 to -0.02 | 55% | 0 to 85 |  |
| **Mental health condition** |  |  |  |  |  |  | 0.960 |
| Formal diagnosis of a psychiatric disorder | 14 | 1100 | -0.71 | -1.11 to -0.31 | 87% | 80 to 92 |  |
| Psychological distress without a diagnosis | 7 | 270 | -0.70 | -1.07 to -0.32 | 51% | 0 to 79 |  |
| **Intervention** |  |  |  |  |  |  | < 0.001 |
| EMDR | 4 | 198 | -1.15 | -2.36 to 0.05 | 92% | 82 to 96 |  |
| NET | 5 | 379 | -0.05 | -0.26 to 0.15 | 0% | 0 to 79 |  |
| CBT | 7 | 298 | -0.95 | -1.63 to -0.26 | 84% | 68 to 92 |  |
| Other** | 5 | 495 | -0.68 | -0.86 to -0.49 | 0% | 0 to 79 |  |
| **Level of intervention** |  |  |  |  |  |  | 0.120 |
| Individual | 17 | 1189 | -0.78 | -1.14 to -0.43 | 85% | 78 to 90 |  |
| Group | 4 | 181 | -0.36 | -0.76 to 0.04 | 36% | 0 to 78 |  |
| **Study quality** |  |  |  |  |  |  | 0.110 |
| Low RoB | 4 | 193 | -1.45 | -2.50 to -0.39 | 88% | 73 to 95 |  |
| High RoB | 17 | 1177 | -0.55 | -0.86 to -0.24 | 81% | 70 to 80 |  |
| Abbreviations: N: number; SMD: Standardized Mean Difference; CI: Confidence Interval; PTSD: Post-Traumatic Stress Disorder; WL: Waiting list; TAU: Treatment as usual; EMDR: Eye Movement Desensitization and Reprocessing ; NET: Narrative Exposure Therapy; CBT: Cognitive-Behavioral Therapy.  Legend:  * Negative values favor active interventions.  ** Other: includes the following interventions: Interpersonal Psychotherapy; Need-satisfaction intervention; Writing for recovering; Common Elements Treatment Approach; Culture-Sensitive Oriented Peer. | | | | | | | |

**Subgroup analyses of depressive outcome (post-treatment)**

| **Meta-analysis** | **Comparisons (N)** | **Patients (N)** | **SMD*** | **95% CI** | ***I*²** | **95% CI** | ***P*** |
| --- | --- | --- | --- | --- | --- | --- | --- |
| Overall depressive outcomes |  |  |  |  |  |  |  |
| All studies | 12 | 844 | -1.02 | -1.52 to -0.51 | 89% | 82 to 93 |  |
| **Subgroup analyses** |  |  |  |  |  |  |  |
| **Country** |  |  |  |  |  |  | < 0.001 |
| High income | 7 | 311 | -0.22 | -0.48 to 0.04 | 16% | 0 to 60 |  |
| Upper, lower middle, low income | 5 | 533 | -2.05 | -3.05 to -1.06 | 93% | 86 to 96 |  |
| **Control condition** |  |  |  |  |  |  | 0.230 |
| TAU or no treatment | 3 | 105 | -2.28 | -5.78 to 1.21 | 97% | 93 to 98 |  |
| WL | 7 | 667 | -0.92 | -1.35 to -0.49 | 78% | 55 to 90 |  |
| Psychological placebo | 2 | 72 | -0.25 | -1.02 to 0.53 | 35% | NA |  |
| **Age** |  |  |  |  |  |  | < 0.001 |
| Adult | 11 | 803 | -0.68 | -1.05 to -0.31 | 77% | 59 to 87 |  |
| Children and mixed | 1 | 41 | -7.07 | -8.79 to -5.34 | NA | NA |  |
| **Mental health condition** |  |  |  |  |  |  | 0.090 |
| Formal diagnosis of a psychiatric disorder | 8 | 693 | -0.62 | -1.07 to -0.16 | 82% | 66 to 91 |  |
| Psychological distress without a diagnosis | 4 | 151 | -2.31 | -4.22 to -0.39 | 95% | 89 to 97 |  |
| **Intervention** |  |  |  |  |  |  | 0.110 |
| EMDR | 4 | 199 | -0.91 | -1.79 to -0.03 | 86% | 64 to 94 |  |
| NET | 2 | 117 | -0.18 | -0.56 to 0.20 | 0% | NA |  |
| CBT | 3 | 122 | -0.52 | -1.41 to 0.38 | 59% | 0 to 88 |  |
| Other** | 3 | 406 | -2.96 | -5.65 to -0.28 | 96% | 91 to 98 |  |
| **Level of intervention** |  |  |  |  |  |  | 0.110 |
| Individual | 11 | 834 | -1.09 | -1.62 to -0.56 | 89% | 83 to 93 |  |
| Group | 1 | 10 | 0.00 | -1.24 to 1.24 | NA | NA |  |
| **Study quality** |  |  |  |  |  |  | 0.370 |
| Low RoB | 2 | 129 | -0.62 | -1.57 to 0.33 | 78% | NA |  |
| High RoB | 10 | 715 | -1.14 | -1.75 to -0.52 | 90% | 83 to 94 |  |
| Abbreviations: N: number; SMD: Standardized Mean Difference; CI: Confidence Interval; PTSD: Post-Traumatic Stress Disorder; WL: Waiting list; TAU: Treatment as usual; EMDR: Eye Movement Desensitization and Reprocessing ; NET: Narrative Exposure Therapy; CBT: Cognitive-Behavioral Therapy.  Legend:  * Negative values favor active interventions.  ** Other: includes the following interventions: Interpersonal Psychotherapy; Need-satisfaction intervention; Common Elements Treatment Approach. | | | | | | | |

**Subgroup analyses of anxiety outcome (post-treatment)**

| **Meta-analysis** | **Comparisons (N)** | **Patients (N)** | **SMD*** | **95% CI** | ***I*²** | **95% CI** | ***P*** |
| --- | --- | --- | --- | --- | --- | --- | --- |
| Overall anxiety outcomes |  |  |  |  |  |  |  |
| All studies | 11 | 815 | -1.05 | -1.55 to -0.56 | 87% | 79 to 92 |  |
| **Subgroup analyses** |  |  |  |  |  |  |  |
| **Country** |  |  |  |  |  |  | 0.840 |
| High income | 7 | 226 | -1.12 | -1.80 to -0.45 | 87% | 76 to 92 |  |
| Upper, lower middle, low income | 2 | 445 | -0.99 | -2.08 to 0.10 | 95% | NA |  |
| **Control condition** |  |  |  |  |  |  | 0.080 |
| TAU or no treatment | 3 | 74 | -2.35 | -4.15 to -0.56 | 86% | 59 to 95 |  |
| WL | 6 | 669 | -0.71 | -1.18 to -0.24 | 84% | 66 to 92 |  |
| Psychological placebo | 2 | 72 | -0.34 | -0.81 to 0.12 | 0% | NA |  |
| **Age** |  |  |  |  |  |  | 0.001 |
| Adult | 10 | 733 | -1.19 | -1.73 to -0.65 | 87% | 79 to 92 |  |
| Children and mixed | 1 | 82 | -0.02 | -0.46 to 0.41 | NA | NA |  |
| **Mental health condition** |  |  |  |  |  |  | 0.520 |
| Formal diagnosis of a psychiatric disorder | 10 | 785 | -1.09 | -1.62 to -0.56 | 89% | 81 to 93 |  |
| Psychological distress without a diagnosis | 1 | 30 | -0.78 | -1.56 to 0.01 | NA | NA |  |
| **Intervention** |  |  |  |  |  |  | 0.110 |
| EMDR | 3 | 170 | -0.86 | -1.82 to 0.11 | 85% | 57 to 95 |  |
| NET | 0 |  |  |  | NA | NA |  |
| CBT | 7 | 298 | -1.36 | -2.25 to -0.47 | 90% | 81 to 94 |  |
| Other** | 1 | 347 | -0.45 | -0.66 to -0.24 | NA | NA |  |
| **Level of intervention** |  |  |  |  |  |  | 0.001 |
| Individual | 9 | 723 | -1.25 | -1.82 to -0.68 | 89% | 81 to 93 |  |
| Group | 2 | 92 | -0.08 | -0.49 to 0.33 | 0% | NA |  |
| **Study quality** |  |  |  |  |  |  | 0.200 |
| Low RoB | 3 | 164 | -2.22 | -4.54 to 0.10 | 95% | 90 to 98 |  |
| High RoB | 8 | 651 | -0.69 | -1.12 to -0.25 | 77% | 54 to 88 |  |
| Abbreviations: N: number; SMD: Standardized Mean Difference; CI: Confidence Interval; PTSD: Post-Traumatic Stress Disorder; WL: Waiting list; TAU: Treatment as usual; EMDR: Eye Movement Desensitization and Reprocessing ; NET: Narrative Exposure Therapy; CBT: Cognitive-Behavioral Therapy.  Legend:  * Negative values favor active interventions.  ** Other: includes the following interventions: Common Elements Treatment Approach. | | | | | | | |

**ANALYSES OF SECONDARY OUTCOMES**

**Secondary outcomes: Other clinical conditions, post-treatment**

**
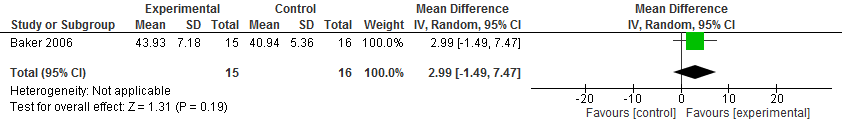
**

**Secondary outcomes: Functioning, post-treatment**

**
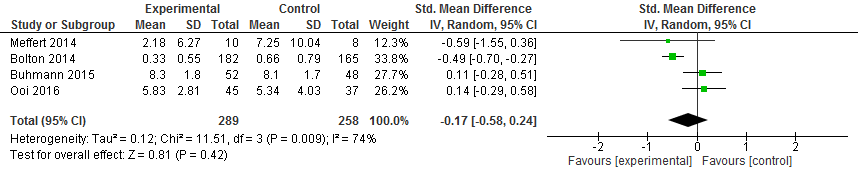
**

**Secondary outcomes: Quality of life, post-treatment**

**
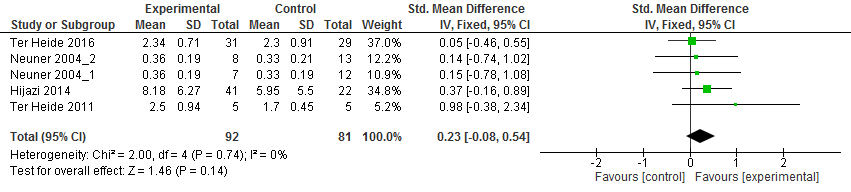
**

**Secondary outcomes: PTSD, follow-up**

**
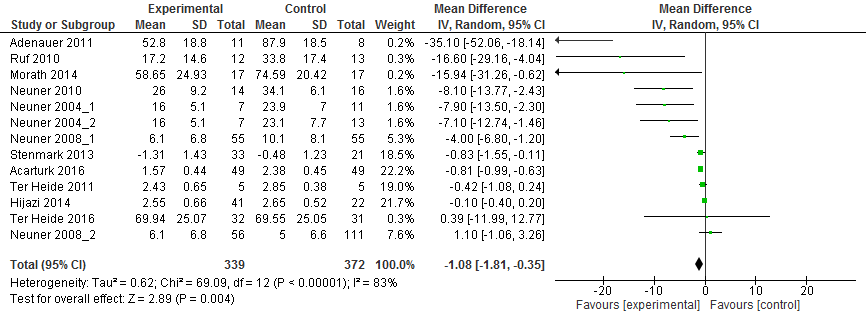
**

**Secondary outcomes: Depression, follow-up**

**
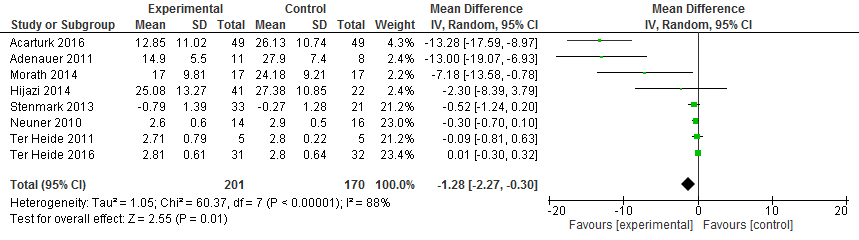
Secondary outcomes: Anxiety, follow-up**

**
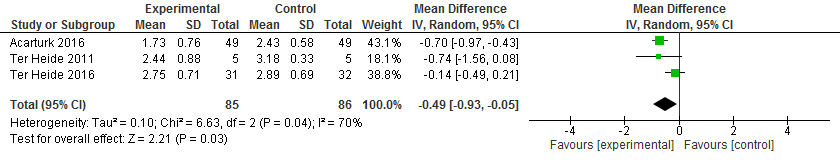
**

**Secondary outcomes: Functioning, follow-up**

**
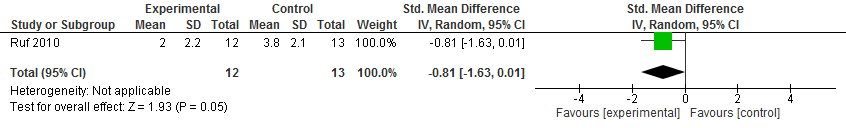
**

**Secondary outcomes: Quality of life, follow-up**

**
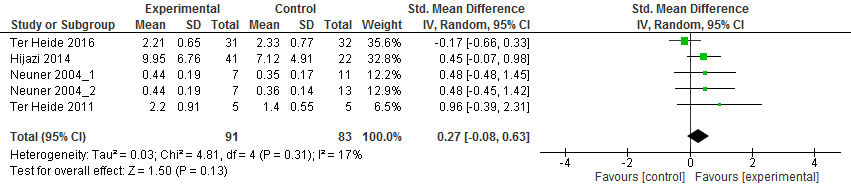
**

**Secondary outcomes: Dropout rates**

**
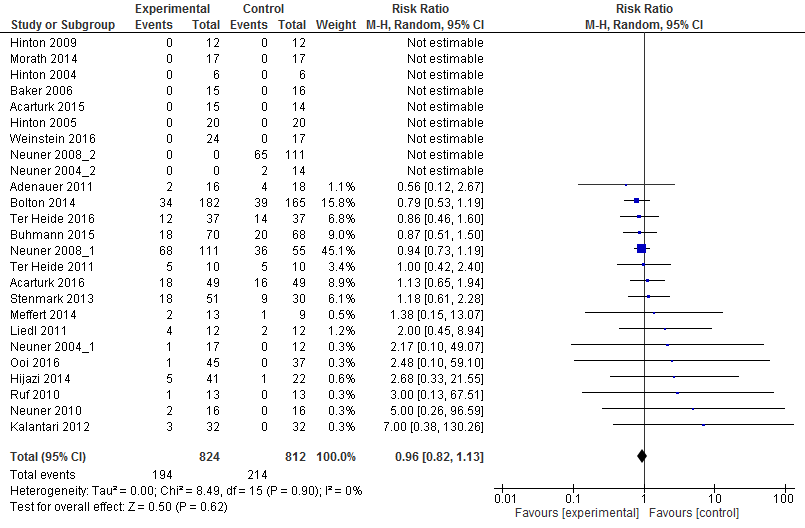
**

**FOREST PLOTS OF SUBGROUP ANALYSES**

**PTSD, post-treatment, by country income level**

**
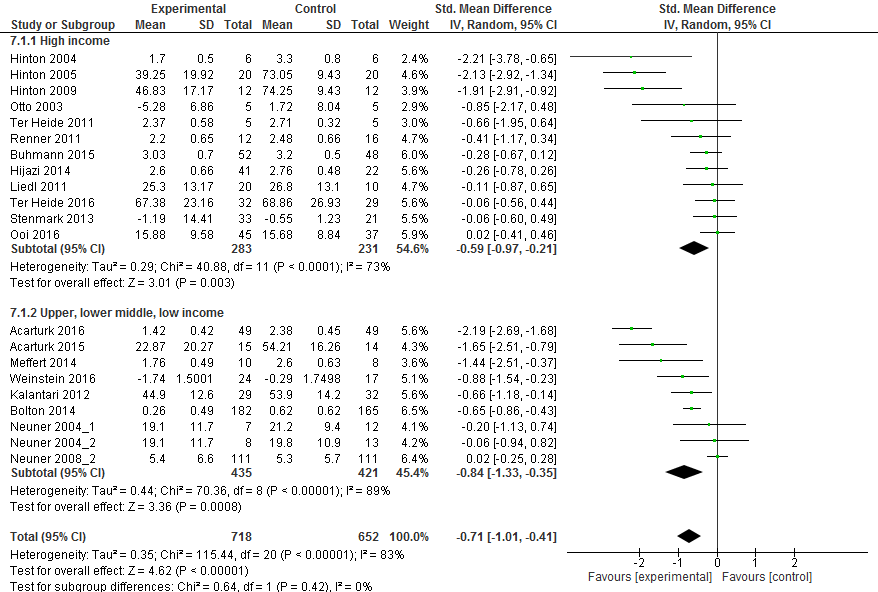
**

**Depression, post-treatment, by country income level**

**
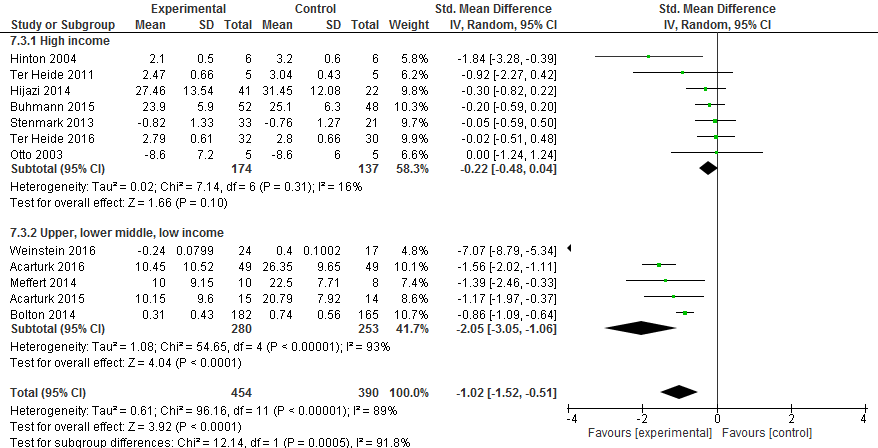
**

**Anxiety, post-treatment, by country income level**

**
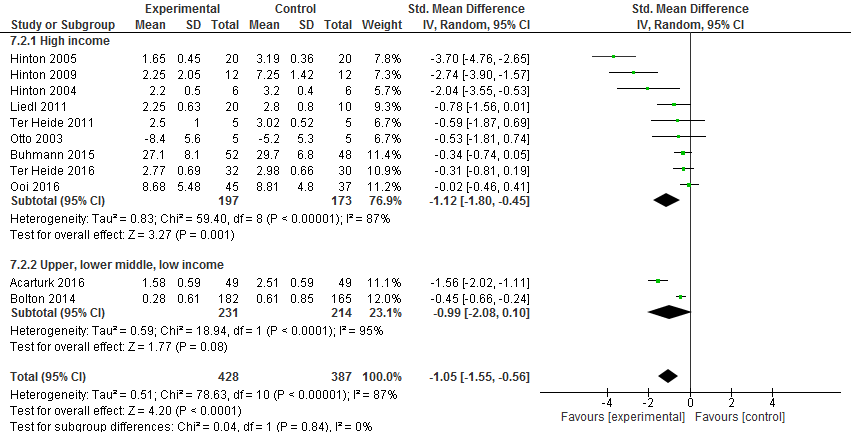
**

**PTSD, post-treatment, by type of control condition**

**
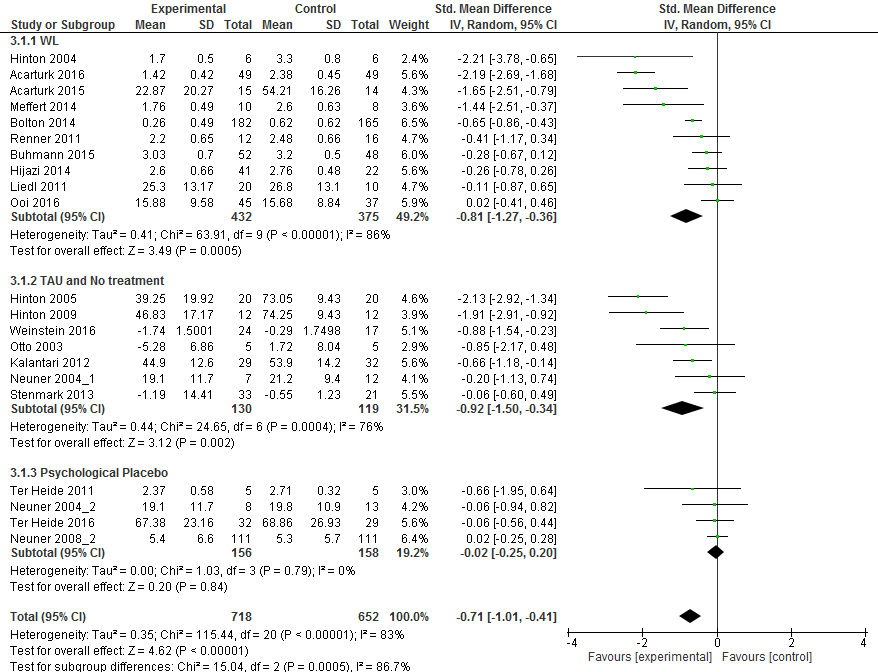
**

**Depression, post-treatment, by type of control condition**

**
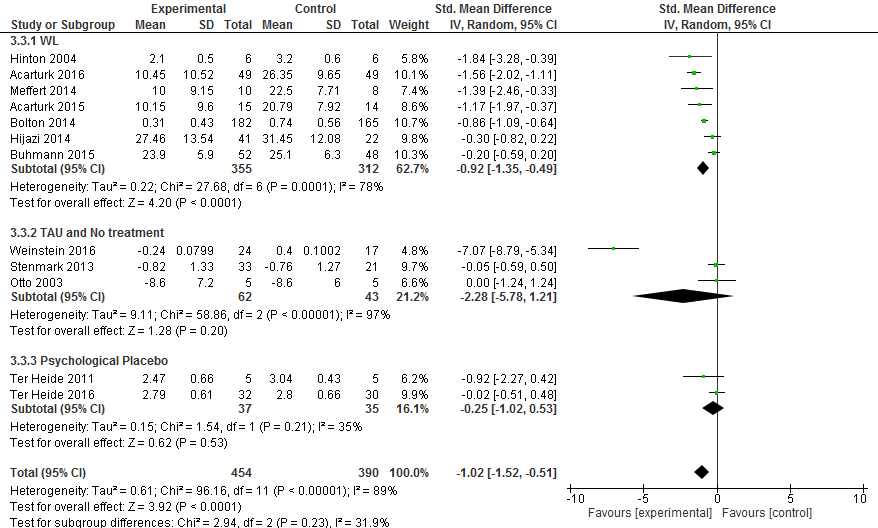
**

**Anxiety, post-treatment, by type of control condition**

**
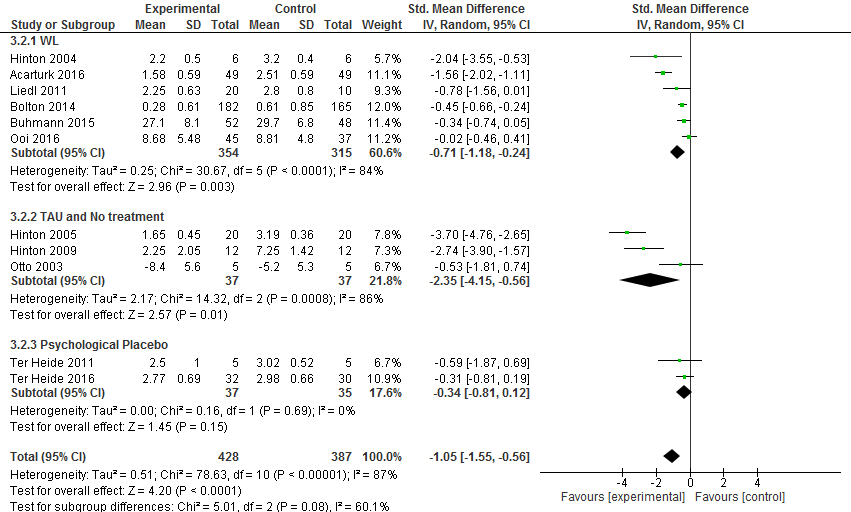
**

**PTSD, post-treatment, by age**

**
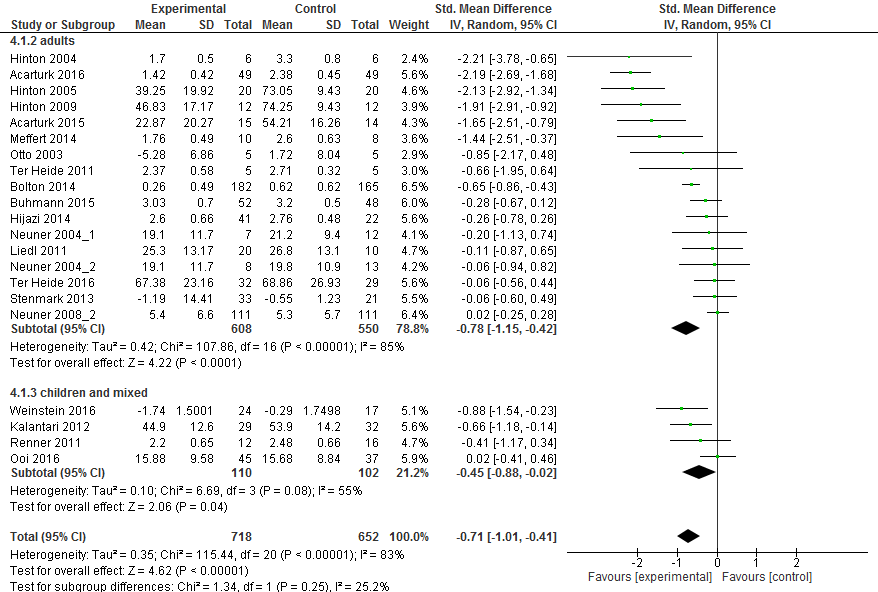
**

**Depression, post-treatment, by age**

**
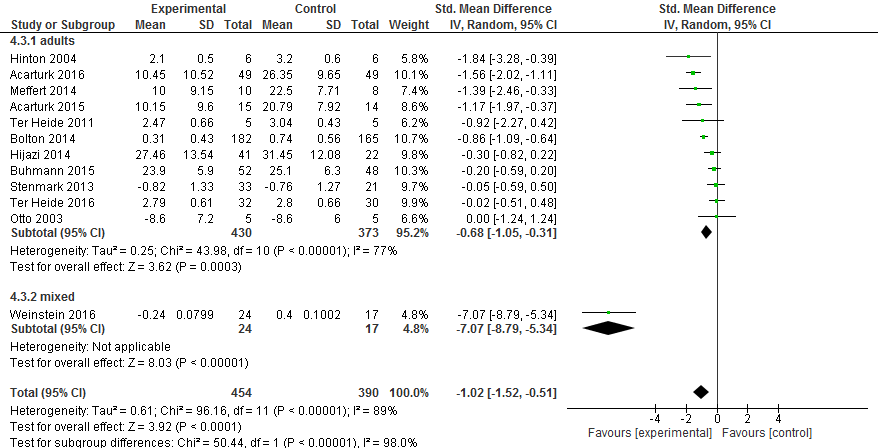
**

**Anxiety, post-treatment, by age**

**
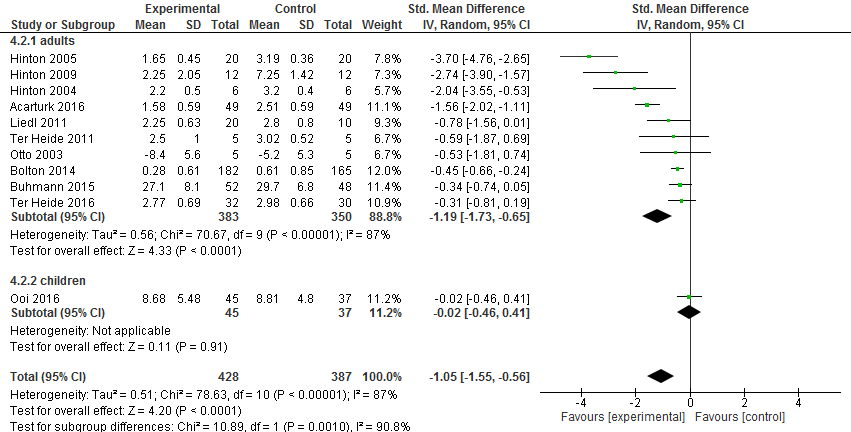
**

**PTSD, post-treatment, by mental health condition**

**
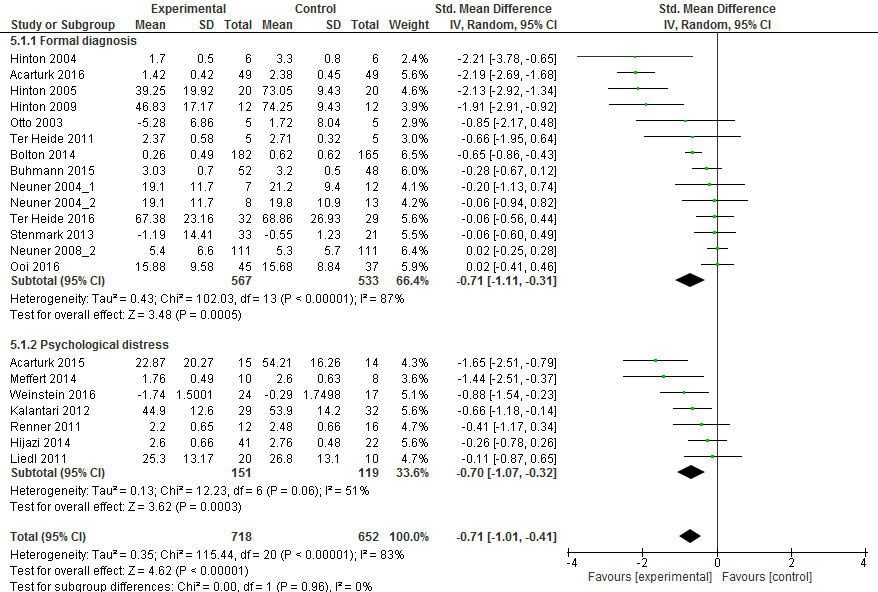
**

**Depression, post-treatment, by mental health condition**

**
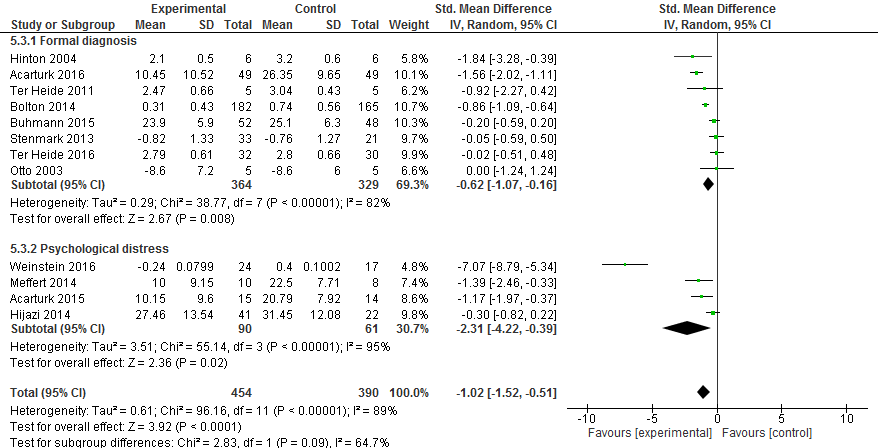
**

**Anxiety, post-treatment, by mental health condition**

**
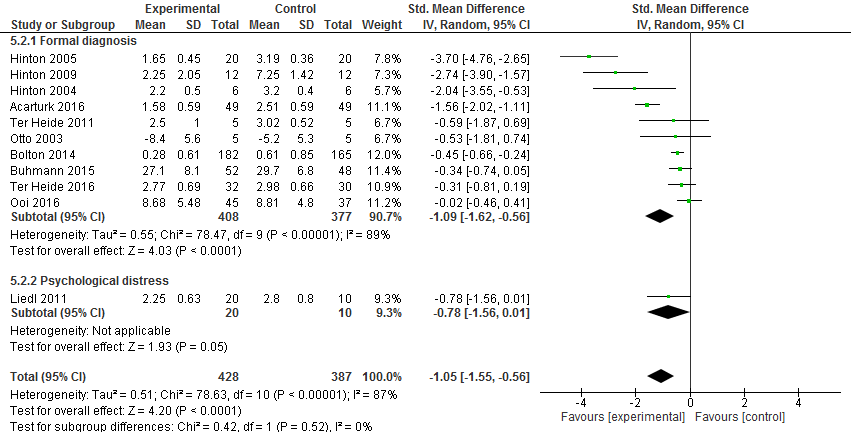
**

**PTSD, post-treatment, by intervention**

**
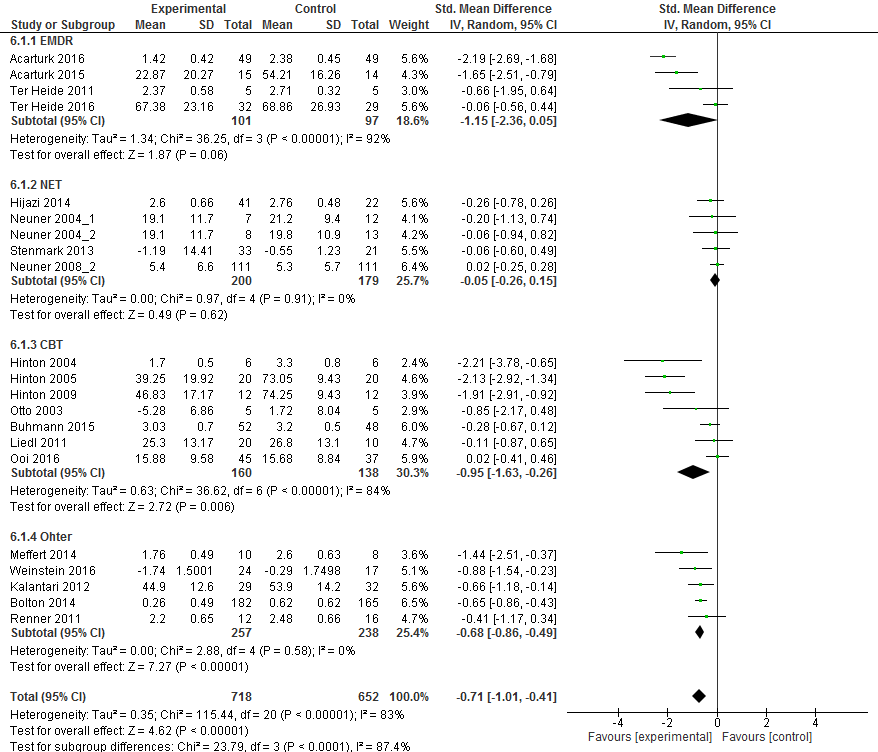
**

**Depression, post-treatment, by intervention**

**
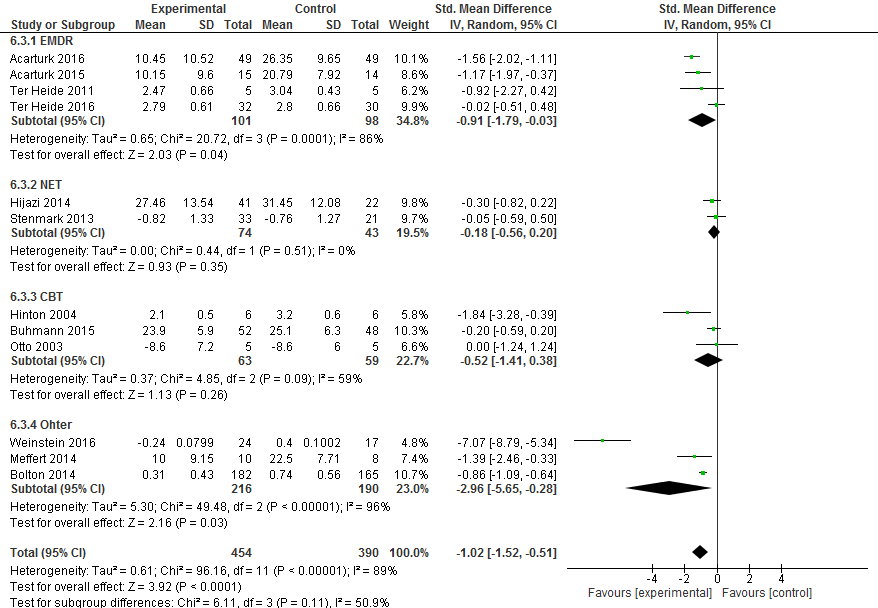
**

**Anxiety, post-treatment, by intervention**

**
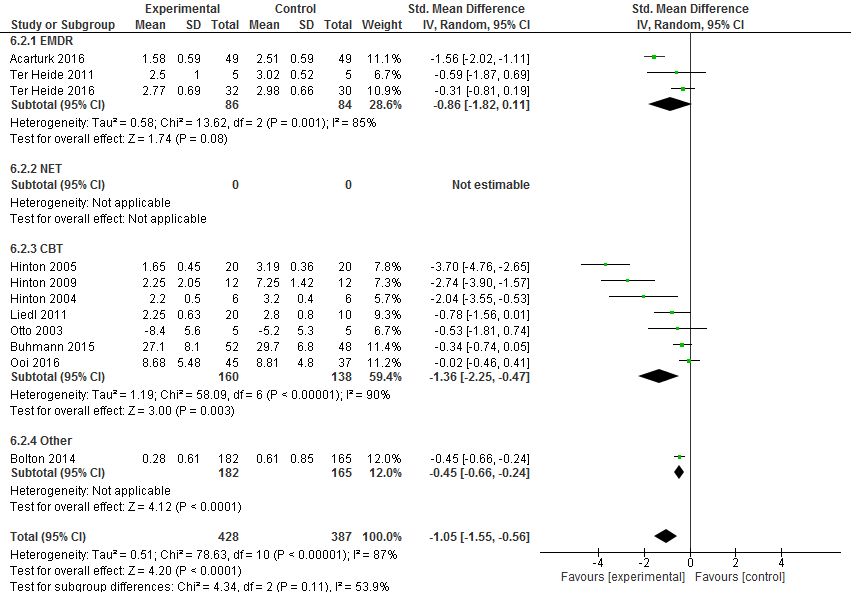
**

**PTSD, post-treatment, by delivery modality**


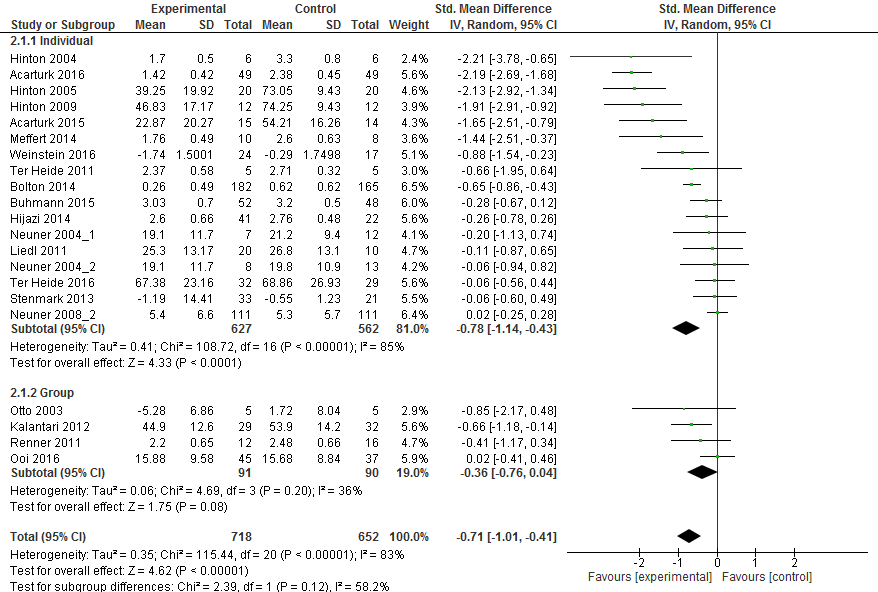


**Depression, post-treatment, by delivery modality**

**
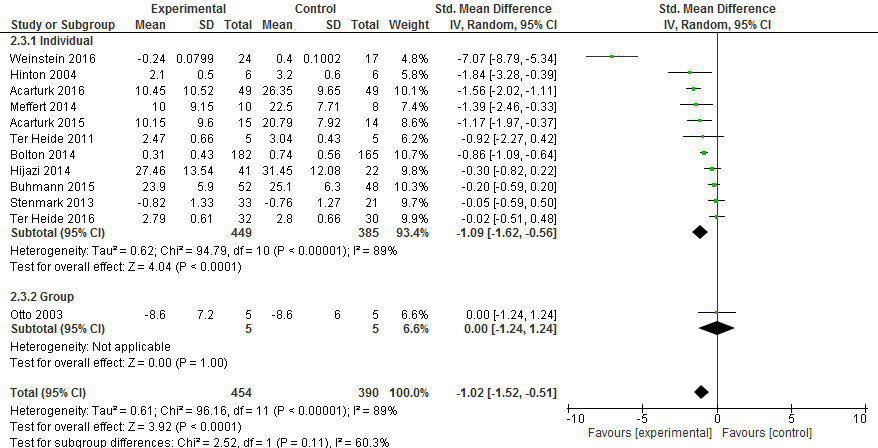
**

**Anxiety, post-treatment, by delivery modality**


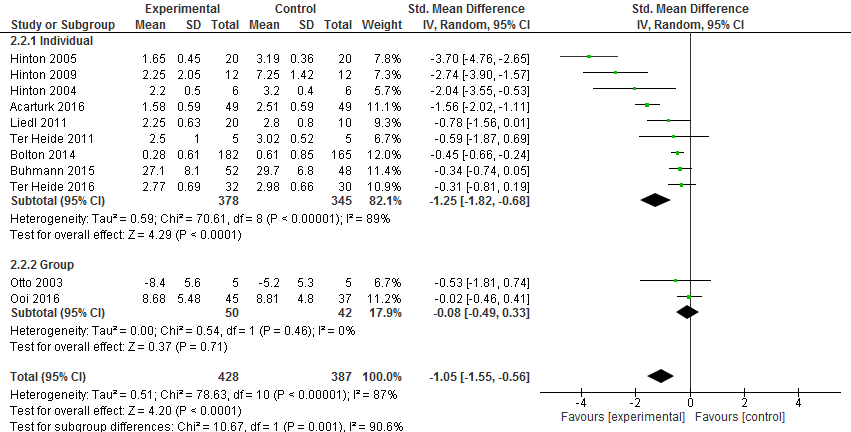


**PTSD, post-treatment, by study quality**

**
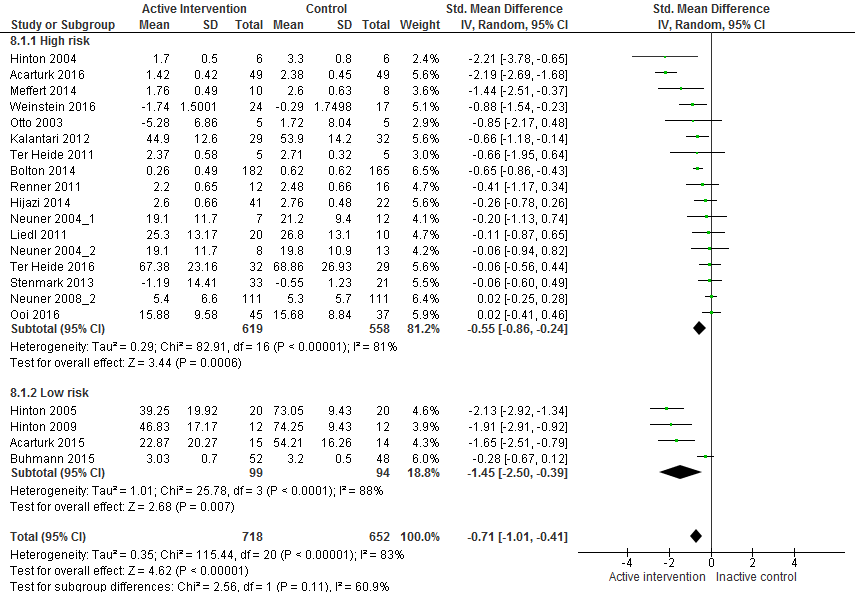
**

**Depression, post-treatment, by study quality**

**
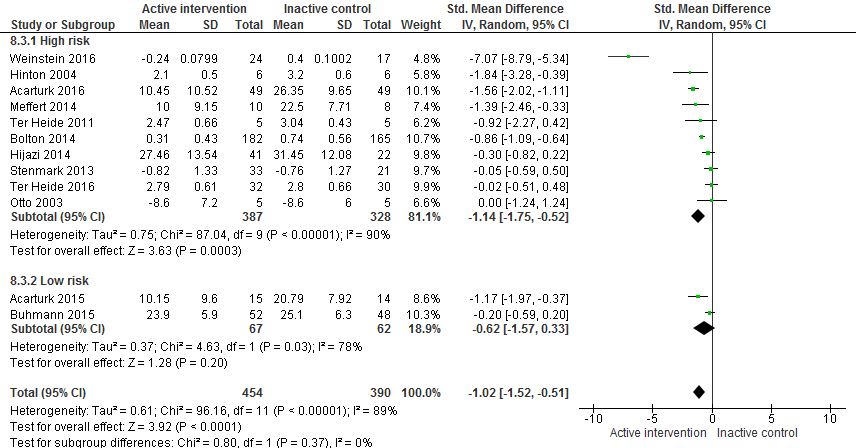
**

**Anxiety, post-treatment, by study quality**

**
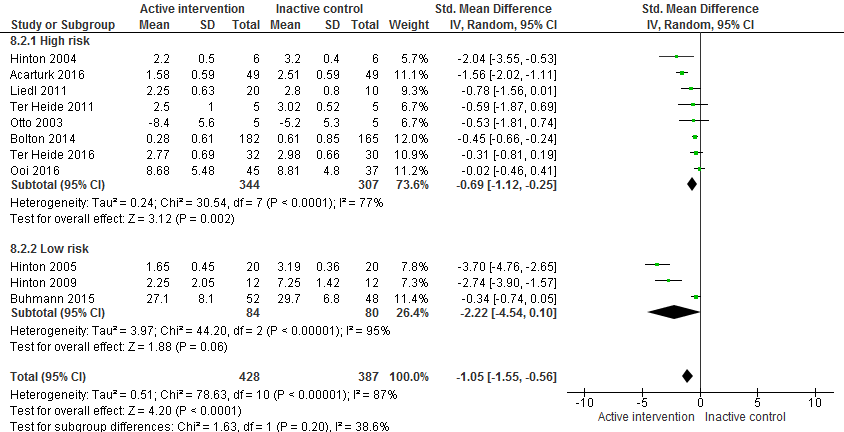
**

**CONVERSION OF PRIMARY OUTCOME RESULTS FROM SMDS INTO ORS AND CORRESPONDING VALUES OF NNTS**

**(assuming different rates of clinically significant PTSD, depression and anxiety symptoms in individuals receiving no treatment)**

| Outcome | SMD | OR | NNT | | | | GRADE |
| --- | --- | --- | --- | --- | --- | --- | --- |
|  |  |  | PEER 60% | PEER 40% | PEER 20% | PEER 10% |  |
| PTSD | -0.71  (-1.01 to -0.41) | 0.26  (0.15 to 0.47) | 3 | 4 | 7 | 14 | MODERATE |
| Depression | -1.02  (-1.52 to -0.51) | 0.14  (0.05 to 0.36) | 2 | 3 | 6 | 12 | MODERATE |
| Anxiety | -1.05  (-1.55 to -0.56) | 0.15  (0.06 to 0.39) | 2 | 3 | 6 | 12 | MODERATE |
| Legend: SMD = standardised mean difference; OR = odds ratio; NNT = number needed to treat;  PEER = patient expected event rate (clinically significant symptoms at study endpoint). | | | | | | | |

**GRADE Evidence Profiles**

**Question**: Are psychosocial interventions compared to inactive control beneficial for asylum seekers and refugees?

**Setting**: High, middle and low income countries.

| **Certainty assessment** | | | | | | | **№ of patients** | | **Effect** | | **Certainty** | **Importance** |
| --- | --- | --- | --- | --- | --- | --- | --- | --- | --- | --- | --- | --- |
| **№ of studies** | **Study design** | **Risk of bias** | **Inconsistency** | **Indirectness** | **Imprecision** | **Other considerations** | **ALL PSYCHOSOCIAL INTERVENTIONS** | **INACTIVE** | **Relative (95% CI)** | **Absolute (95% CI)** |  |  |
| PTSD (post-treatment) | | | | | | | | | | | | |
| 20 | randomised trials | serious ^a^ | serious ^b^ | not serious | not serious | strong association ^c^ | 718 | 652 | - | SMD **0.71 lower** (1.01 lower to 0.41 lower) | ⨁⨁⨁◯ MODERATE | CRITICAL |
| Anxiety (post-treatment) | | | | | | | | | | | | |
| 11 | randomised trials | serious ^d^ | serious ^b^ | not serious | not serious | strong association ^e^ | 428 | 387 | - | SMD **1.05 lower** (1.55 lower to 0.56 lower) | ⨁⨁⨁◯ MODERATE | CRITICAL |
| Depression (post-treatment) | | | | | | | | | | | | |
| 12 | randomised trials | serious ^f^ | serious ^b^ | not serious | not serious | strong association ^g^ | 454 | 390 | - | SMD **1.02 lower** (1.52 lower to 0.51 lower) | ⨁⨁⨁◯ MODERATE | CRITICAL |

**CI:** Confidence interval; **SMD:** Standardised mean difference; **MD:** Mean difference; **OR:** Odds ratio

#### Explanations

a. Most studies were at high risk of bias in two or three items of the Cochrane risk of bias tool. All studies had methodological shortcomings related to masking of participants and personnel.

b. Visual investigation of forest plot suggests some heterogeneity (higher than 75%), however we decided to downgrade by one level only, bacause of an expected result. A lot of differences exist in this population and the assessment of all existing variables is difficult.

c. The SMD of -0.71 corresponds to an OR of 0.26 (0.15 to 0.47) which suggests a large effect.

d. Almost 30% of the studies were at high risk of bias in one of following two items of the Cochrane risk of bias tool: blinding of outcome assessors; dropout rate. Moreover, all studies had methodological shortcomings related to masking of participants and personnel.

e. The SMD of -1.05 corresponds to an OR of 0.15 (0.06 to 0.39) which suggests a large effect.

f. More than 10% of the studies were at high risk of bias in one of the following two items of the Cochrane risk of bias tool: blinding of outcome assessors; dropout rate. Moreover, all studies had methodological shortcomings related to masking of participants and personnel.

g. The SMD of -1.02 corresponds to an OR of 0.14 (0.05 to 0.36) which suggests a large effect.

**FUNNEL PLOT OF THE PRIMARY OUTCOME: PTSD SYMPTOMS POST TREATMENT**

According to Duval and Tweedie’s trim and fill procedure there are relatively few studies falling toward the right of the mean effect (see funnel plot below), raising a concern that these right-hand studies may actually exist, and are missing from the analysis. Duval and Tweedie’s procedure allows to impute these studies to determine where the missing studies are likely to fall (black dots), add them to the analysis, and then re-compute the combined effect, as reported in the Funnel plot below.

**
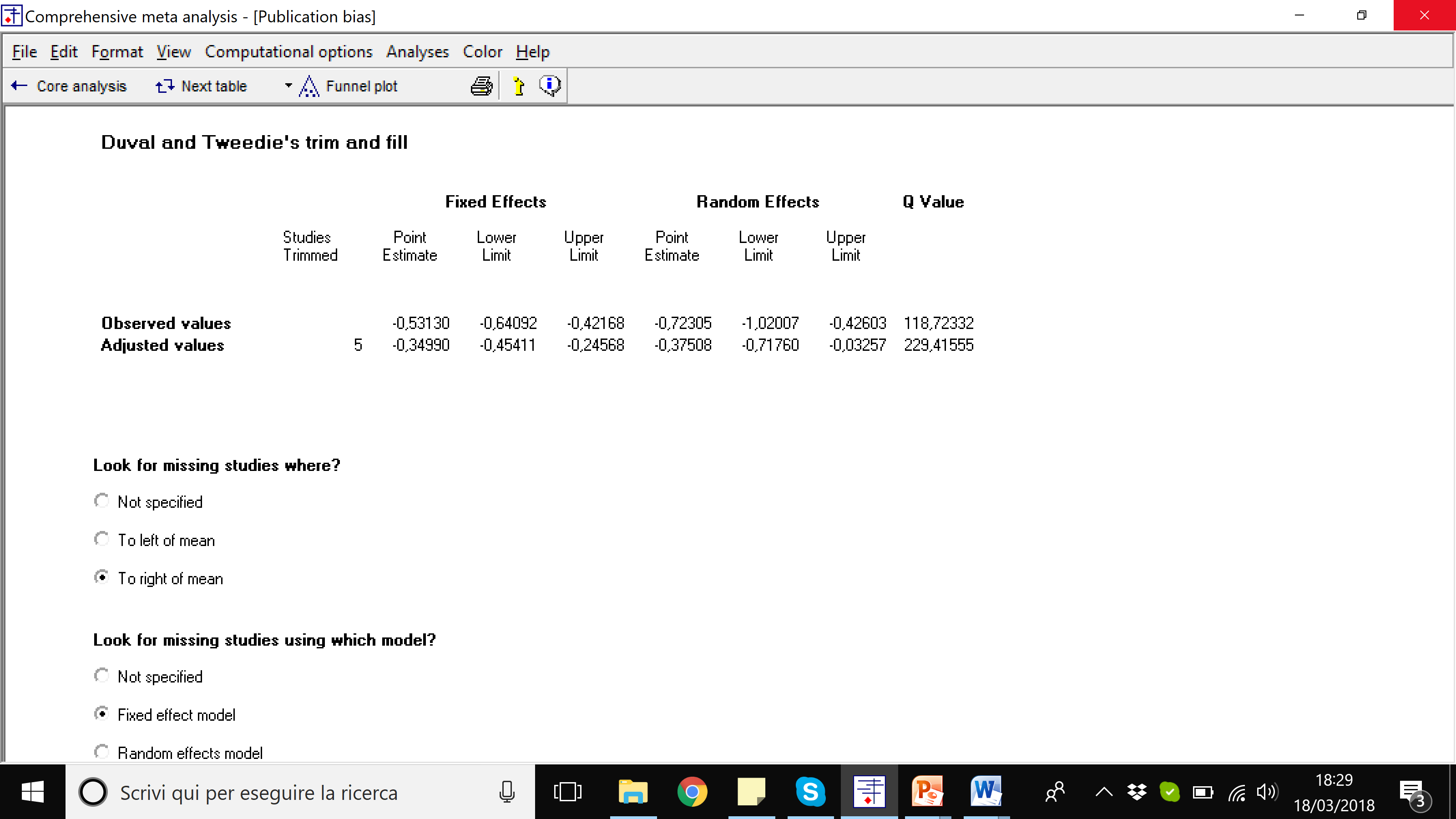
**

**
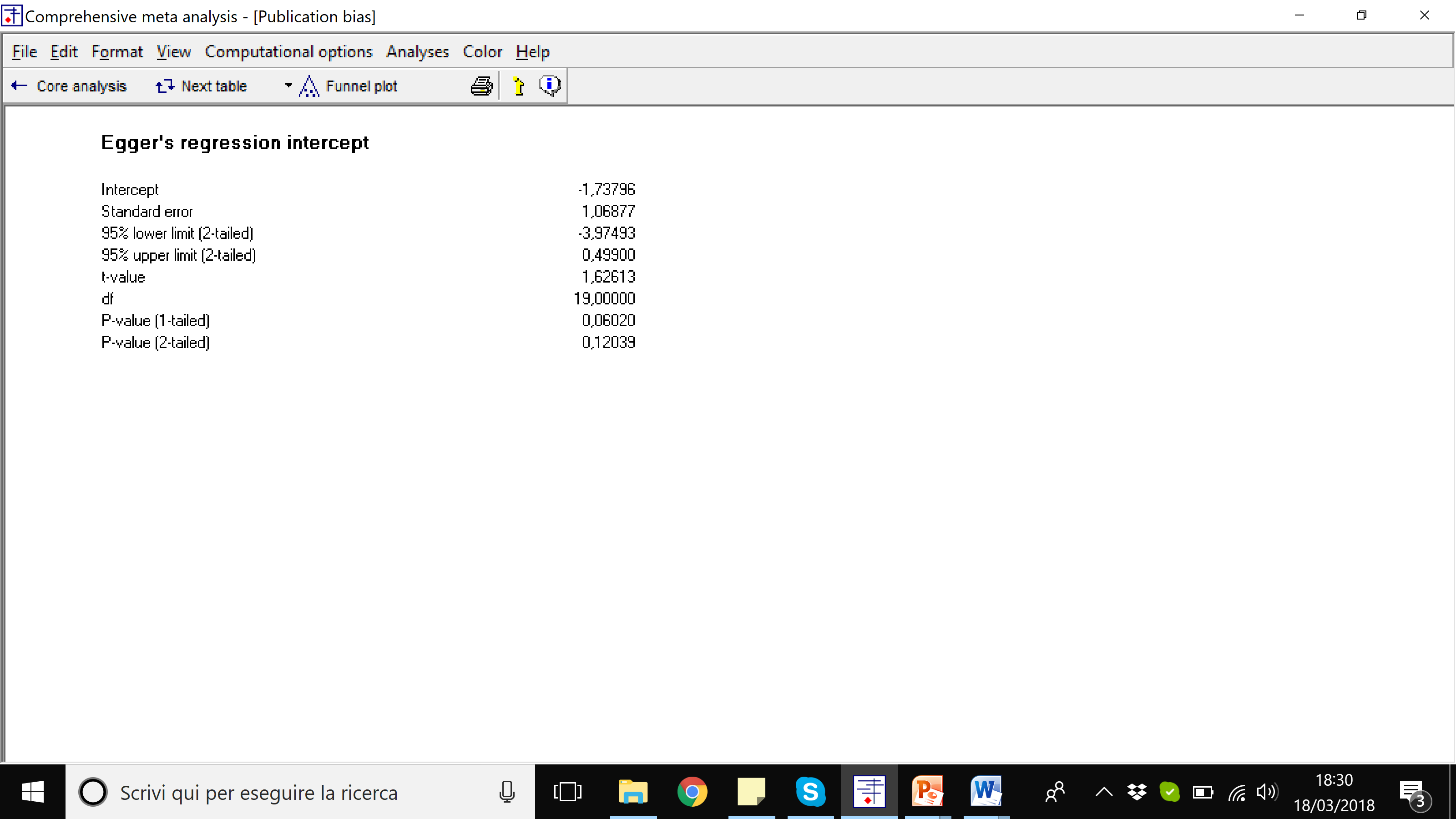
**

**IMPACT OF EACH STUDY ON THE POOLED EFFECT**

Consecutively removal of each study as a possible outlier to test what the impact is on the combined effect

**PRISMA checklist**

| **Section/topic** | **#** | **Checklist item** | **Reported on page #** |
| --- | --- | --- | --- |
| **TITLE** | | |  |
| Title | 1 | Identify the report as a systematic review, meta-analysis, or both. | 1 |
| **ABSTRACT** | | |  |
| Structured summary | 2 | Provide a structured summary including, as applicable: background; objectives; data sources; study eligibility criteria, participants, and interventions; study appraisal and synthesis methods; results; limitations; conclusions and implications of key findings; systematic review registration number. | 3-4 |
| **INTRODUCTION** | | |  |
| Rationale | 3 | Describe the rationale for the review in the context of what is already known. | 5 |
| Objectives | 4 | Provide an explicit statement of questions being addressed with reference to participants, interventions, comparisons, outcomes, and study design (PICOS). | 6 |
| **METHODS** | | |  |
| Protocol and registration | 5 | Indicate if a review protocol exists, if and where it can be accessed (e.g., Web address), and, if available, provide registration information including registration number. | 7 |
| Eligibility criteria | 6 | Specify study characteristics (e.g., PICOS, length of follow-up) and report characteristics (e.g., years considered, language, publication status) used as criteria for eligibility, giving rationale. | 7-8 |
| Information sources | 7 | Describe all information sources (e.g., databases with dates of coverage, contact with study authors to identify additional studies) in the search and date last searched. | 7 |
| Search | 8 | Present full electronic search strategy for at least one database, including any limits used, such that it could be repeated. | Appendix |
| Study selection | 9 | State the process for selecting studies (i.e., screening, eligibility, included in systematic review, and, if applicable, included in the meta-analysis). | 7 |
| Data collection process | 10 | Describe method of data extraction from reports (e.g., piloted forms, independently, in duplicate) and any processes for obtaining and confirming data from investigators. | 9 |
| Data items | 11 | List and define all variables for which data were sought (e.g., PICOS, funding sources) and any assumptions and simplifications made. | 9 |
| Risk of bias in individual studies | 12 | Describe methods used for assessing risk of bias of individual studies (including specification of whether this was done at the study or outcome level), and how this information is to be used in any data synthesis. | 9-10 |
| Summary measures | 13 | State the principal summary measures (e.g., risk ratio, difference in means). | 10-11 |
| Synthesis of results | 14 | Describe the methods of handling data and combining results of studies, if done, including measures of consistency (e.g., I^2^) for each meta-analysis. | 12-15 |

*From:*  Moher D, Liberati A, Tetzlaff J, Altman DG, The PRISMA Group (2009). Preferred Reporting Items for Systematic Reviews and Meta-Analyses: The PRISMA Statement. PLoS Med 6(7): e1000097. doi:10.1371/journal.pmed1000097

*For more information, visit: www.prisma-statement.or*
