# Supplementary material for: Efficacy and acceptability of psychosocial interventions in asylum seekers and refugees: systematic review and meta-analysis
Source: Epidemiol Psychiatr Sci. 2019 Feb 11;28(4):376–88. doi: 10.1017/S2045796019000027 (PMC6669989; doi:10.1017/S2045796019000027)
Supplement: Supplementary file 1 [file S2045796019000027sup.zip › S2045796019000027sup001/Appendix_2.pdf]

Post-Traumatic Stress Disorder outcomes

Study	mean_exp_PTSD	SD_exp_PTSD	tot_exp_PTSD	mean_cont_PTSD	SD_cont_PTSD	tot_cont_PTSD	income	control	age	diagnosis	treatment
Hinton 2004	1,70	0,50	6	3,30	0,80	6	1	1	1	1	3
Acarturk 2016	1,42	0,42	49	2,38	0,45	49	0	1	1	1	1
Hinton 2005	39,25	19,92	20	73,05	9,43	20	1	2	1	1	3
Hinton 2009	46,83	17,17	12	74,25	9,43	12	1	2	1	1	3
Acarturk 2015	22,87	20,27	15	54,21	16,26	14	0	1	1	0	1
Meffert 2014	1,76	0,49	10	2,60	0,63	8	0	1	1	0	4
Otto 2003	-5,28	6,86	5	1,72	8,04	5	1	2	1	1	3
Kalantari 2012	44,90	12,60	29	53,90	14,20	32	0	2	0	0	4
Ter Heide 2011	2,37	0,58	5	2,71	0,32	5	1	3	1	1	1
Bolton 2014	0,26	0,49	182	0,62	0,62	165	0	1	1	1	4
Renner 2011	2,20	0,65	12	2,48	0,66	16	1	1	0	0	4
Buhmann 2015	3,03	0,70	52	3,20	0,50	48	1	1	1	1	3
Hijazi 2014	2,60	0,66	41	2,76	0,48	22	1	1	1	0	2
Neuner 2004_1	19,10	11,70	7	21,20	9,40	12	0	2	1	1	2
Liedl 2011	25,30	13,17	20	26,80	13,10	10	1	1	1	0	3
Neuner 2004_2	19,10	11,70	8	19,80	10,90	13	0	3	1	1	2
Ter Heide 2016	67,38	23,16	32	68,86	26,93	29	1	3	1	1	1
Stenmark 2013	-1,19	14,41	33	-0,55	1,23	21	1	2	1	1	2
Neuner 2008_2	5,40	6,60	111	5,30	5,70	111	0	3	1	1	2
Ooi 2016	15,88	9,58	45	15,68	8,84	37	1	1	0	1	3
Weinstein 2016	-1,74	1,50	24	-0,29	1,74	17	0	2	0	0	4

Depressive outcomes

Study	mean_exp_depression	SD_exp_depression	tot_exp_depression	mean_cont_depression	SD_cont_depression	tot_cont_depression
Hinton 2004	2,1	0,5	6	3,2	0,6	6
Acarturk 2016	10,45	10,52	49	26,35	9,65	49
Meffert 2014	10	9,15	10	22,5	7,71	8
Acarturk 2015	10,15	9,6	15	20,79	7,92	14
Ter Heide 2011	2,47	0,66	5	3,04	0,43	5
Bolton 2014	0,31	0,43	182	0,74	0,56	165
Hijazi 2014	27,46	13,54	41	31,45	12,08	22
Buhmann 2015	23,9	5,9	52	25,1	6,3	48
Stenmark 2013	-0,82	1,33	33	-0,76	1,27	21
Ter Heide 2016	2,79	0,61	32	2,8	0,66	30
Otto 2003	-8,6	7,2	5	-8,6	6	5
Wenstein 2016	-0,24	0,07	24	0,4	0,1	17

Anxiety outcomes

Study	mean_exp_anxiety	SD_exp_anxiety	tot_exp_anxiety	mean_cont_anxiety	SD_cont_anxiety	tot_cont_anxiety
Hinton 2005	1,65	0,45	20	3,19	0,36	20
Hinton 2009	2,25	2,05	12	7,25	1,42	12
Hinton 2004	2,2	0,5	6	3,2	0,4	6
Liedl 2011	2,25	0,63	20	2,8	0,8	10
Ter Heide 2011	2,5	1	5	3,02	0,52	5
Otto 2003	-8,4	5,6	5	-5,2	5,3	5
Bolton 2014	0,28	0,61	182	0,61	0,85	165
Buhmann 2015	27,1	8,1	52	29,7	6,8	48
Ter Heide 2016	2,77	0,69	32	2,98	0,66	30
Ooi 2016	8,68	5,48	45	8,81	4,8	37
Acarturk 2016	1,58	0,59	49	2,51	0,59	49

Legend

mean	SD	tot	exp
mean= mean scores,post-intervention	SD= Standard Deviation, post-intervention	tot= number of participants	exp= experimental arm

cont	income	control	age
cont= control arm	0= Upper, lower middle, low income<br>1= HIC	1= WL<br>2= no treatment<br>3= TAU	0= children and mixed<br>1= adults

treatment	diagnosis
1= EMDR<br>2= NET<br>3= CBT<br>4= Other	0= symptoms<br>1= formal diagnosis
